# Empirical effect of the Dr LEE Jong-wook Fellowship Program to empower sustainable change for the health workforce in Tanzania: a mixed-methods study

**DOI:** 10.3352/jeehp.2025.22.6

**Published:** 2025-01-20

**Authors:** Masoud Dauda, Swabaha Aidarus Yusuph, Harouni Yasini, Issa Mmbaga, Perpetua Mwambinngu, Hansol Park, Gyeongbae Seo, Kyoung Kyun Oh

**Affiliations:** 1 Department of Sociology and Anthropology, University of Dar es Salaam, Dar es Salaam, Tanzania; 2 Korea Foundation for International Healthcare (KOFIH) Tanzania Office, Dar es Salaam, Tanzania; 3 Department of Human Resources, Ministry of Health, Dodoma, Tanzania; 4 National Blood Transfusion Services, Dar es Salaam, Tanzania; 5 Korea Foundation for International Healthcare (KOFIH), Seoul, Korea; The Catholic University of Korea, Korea

**Keywords:** Health Workforce, Empowerment, Scholarships and Fellowships, Program Evaluation, International Cooperation, Tanzania

## Abstract

**Purpose:**

This study evaluated the Dr LEE Jong-wook Fellowship Program’s impact on Tanzania’s health workforce, focusing on relevance, effectiveness, efficiency, impact, and sustainability in addressing healthcare gaps.

**Methods:**

A mixed-methods research design was employed. Data were collected from 97 out of 140 alumni through an online survey, 35 in-depth interviews, and one focus group discussion. The study was conducted from November to December 2023 and included alumni from 2009 to 2022. Measurement instruments included structured questionnaires for quantitative data and semi-structured guides for qualitative data. Quantitative analysis involved descriptive and inferential statistics (Spearman’s rank correlation, non-parametric tests) using Python ver. 3.11.0 and Stata ver. 14.0. Thematic analysis was employed to analyze qualitative data using NVivo ver. 12.0.

**Results:**

Findings indicated high relevance (mean=91.6, standard deviation [SD]=8.6), effectiveness (mean=86.1, SD=11.2), efficiency (mean=82.7, SD=10.2), and impact (mean=87.7, SD=9.9), with improved skills, confidence, and institutional service quality. However, sustainability had a lower score (mean=58.0, SD=11.1), reflecting challenges in follow-up support and resource allocation. Effectiveness strongly correlated with impact (ρ=0.746, P<0.001). The qualitative findings revealed that participants valued tailored training but highlighted barriers, such as language challenges and insufficient practical components. Alumni-led initiatives contributed to knowledge sharing, but limited resources constrained sustainability.

**Conclusion:**

The Fellowship Program enhanced Tanzania’s health workforce capacity, but it requires localized curricula and strengthened alumni networks for sustainability. These findings provide actionable insights for improving similar programs globally, confirming the hypothesis that tailored training positively influences workforce and institutional outcomes.

## Graphical abstract

[Fig f3-jeehp-22-06]

## Introduction

### Background

Human resources for health (HRH) form the backbone of effective healthcare systems, particularly in low-and middle-income countries (LMICs) such as Tanzania, where workforce shortages severely hinder healthcare delivery [1]. Despite ongoing global initiatives to address HRH gaps, Tanzania continues to face critical challenges, including limited training opportunities, high turnover rates, and inadequate capacity-building programs [2]. Previous international training initiatives, such as the World Health Organization Fellowship Program and US’s training programs, have demonstrated short-term benefits by improving individual competencies [3,4]. However, evidence is limited regarding the sustainability and long-term impact of these programs, particularly in LMICs. Addressing this gap is crucial for strengthening health systems and achieving equitable healthcare access. This study focuses on the Dr LEE Jong-wook Fellowship Program (hereinafter referred to as the Fellowship Program), established in 2009 by the Korea Foundation for International Healthcare (KOFIH), to evaluate its impact on Tanzania’s health workforce and health systems. Understanding the Fellowship Program’s relevance, effectiveness, efficiency, impact, and sustainability is essential for informing future capacity-building initiatives globally.

### Objectives

This study evaluated the Fellowship Program’s long-term outcomes on Tanzania’s health workforce by examining its relevance, effectiveness, efficiency, impact, sustainability, and alignment with national priorities, focusing on enhancing competencies and institutional capacity.

## Methods

### Ethics statement

Ethical approval was obtained from the University of Dar es Salaam Research Ethics Committee (UDSM-REC) (approval number: CoSS-SO23166). And informed consent was obtained from the participants.

### Study design

This mixed-methods study assessed the Fellowship Program’s impact through a quantitative survey (online questionnaire) and a qualitative analysis (Supplement 1), which included 30 in-depth interviews (IDIs) with alumni, of whom 6 were also participants in a focus group discussion (FGD). Additionally, 5 more IDIs were conducted with healthcare leaders.

### Setting

The study focused on Tanzania, where the Fellowship Program has been implemented since 2009. Data were collected from November to December 2023 and covered the activities conducted between 2009 and 2022.

### Participants

Participants included all 140 Fellowship Program alumni (2009–2022). Eligibility required program participation and employment in healthcare in Tanzania. Ninety-seven alumni completed surveys, while 30 alumni, 5 healthcare leaders, and 6 FGD participants provided qualitative data. Alumni who were outside the health sector or unreachable were excluded.

### Variables

The terms relevance, effectiveness, efficiency, impact, and sustainability are aligned with the definitions of the Organisation for Economic Co-operation and Development-Development Assistance Committee (OECD-DAC) [5]. Table 1 shows more details on each criterion’s specific questions and definition.

### Data sources/measurement

Quantitative data were collected using structured online questionnaires consisting of Likert-scale and open-ended questions (Dataset 1). The internal consistency reliability was 0.895 for the effectiveness construct, 0.897 for the impact construct, and 0.779 for the sustainability construct. However, the values for the relevance construct and the efficiency contract were 0.675 and 0. 687, respectively. Despite this, they can still be tolerated as they are somewhat close to the acceptable threshold of 0.70 (Table 2). Qualitative data were collected via semi-structured interview guides designed to explore the themes of relevance, effectiveness, efficiency, impact, and sustainability (Dataset 2). IDIs and the FDG were conducted in Swahili, and the interviews were audio-recorded, transcribed verbatim, and translated into English where necessary.

### Bias

Selection bias was reduced by diversifying participants, and recall bias was minimized by focusing on recent experiences, conducting triangulation and cross-validation among participants, and reducing interviewer bias through standardized guides and independent coding.

### Study size

The total cumulative number of the Fellowship Program’s participants was 165, though the total number of alumni was 140. Ninety-seven out of 140 alumni completed the online survey (69% response rate). Qualitative data included 30 IDIs with the alumni, of whom 6 were also participants in the FGD. Five more IDIs were conducted with healthcare leaders who were not alumni, ensuring robust triangulation and validation.

### Statistical methods

**Fig. 1.**
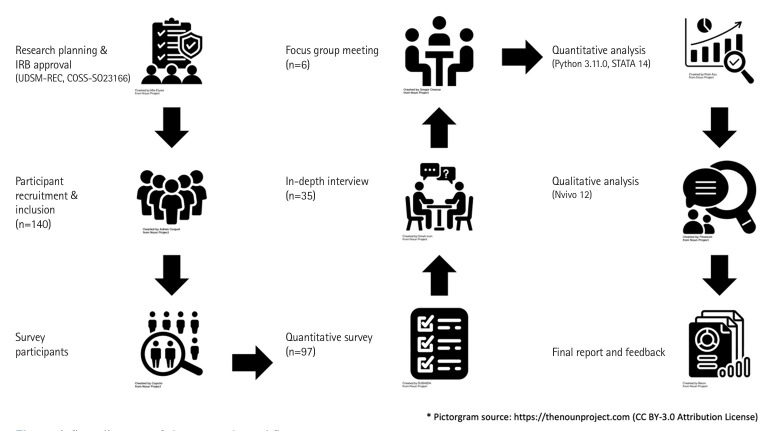
A flow diagram of the research workflow.

Quantitative data were analyzed with Python ver. 3.11.0 (https://www.python.org/) and Stata ver. 14.0 (Stata Corp.) using Spearman’s correlation and non-parametric tests. Qualitative data were thematically analyzed via NVivo ver. 12.0 (Lumivero), with independent coding by 2 researchers (M.D. and H.Y.) and consensus on discrepancies. A research workflow diagram is shown in Fig. 1.

## Results

### General characteristics of the respondents

A total of 97 alumni completed the online survey, supplemented by 35 IDIs and one FGD (Supplement 2). Participants predominantly came from Dar es Salaam, Dodoma, and Pwani (85.6%). Among survey participants, 56.7% were male, and 43.3% female, with an age distribution as follows: 25–34 years, 21.6%; 35–44 years, 43.3%; and 45–54 years, 30.9%. National hospitals represented the largest share of respondents, followed by the Ministry of Health. Half of the participants had clinical training backgrounds. Among 35 interviewees, 60.0% were male and 40.0% female, with 48.6% from Dar es Salaam, 22.9% from Dodoma, and 28.6% from Pwani. Clinical backgrounds were dominant among the participants in the qualitative research (68.6%), while 8.6% were from biomedical engineering and health policy and administration courses. Five participants in the FGD were not alumni (Table 3).

**Fig. 2.**
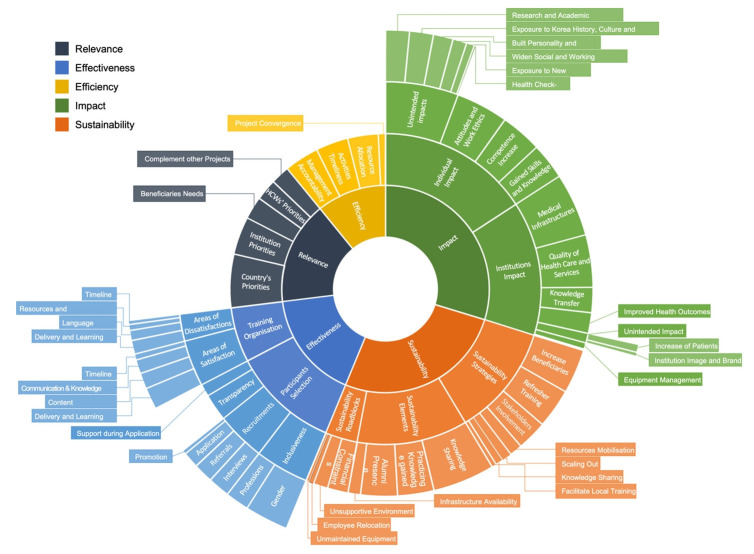
Thematic analysis of the Fellowship Program based on in-depth interviews.

Thematic analysis revealed the following themes: relevance (sub-themes: country’s priorities, institution priorities, beneficiaries’ needs, healthcare workers’ priorities, and complementing other projects), effectiveness (sub-themes: training organization, participant selection), efficiency (sub-themes: management accountability, activities timeliness, resource allocation, project convergence), impact (sub-themes: institutions impact, individual impact), and sustainability (sub-themes: sustainability strategies, sustainability elements, sustainability roadblocks). Fig. 2 provides an overview of these themes.

### Quantitative results

The quantitative analysis showed the Fellowship Program had the highest score for relevance (mean=91.6, standard deviation [SD]=8.6), with effectiveness (mean=86.1, SD=11.2), efficiency (mean=82.7, SD=10.2), and impact (mean=87.7, SD=9.9) also rated positively. However, sustainability had the lowest score (mean=58.0, SD=11.1), highlighting concerns about long-term benefits and outcomes (Table 4).

Spearman rank-order correlation analysis showed significant positive associations across evaluation criteria, with the strongest relationships observed between effectiveness and impact (ρ=0.746, P<0.001) and relevance and effectiveness (ρ=0.688, P<0.001) (Table 5).

The Mann-Whitney U test showed no significant differences, although the Kruskal-Wallis test demonstrated significant differences in perceived training content relevance across age groups (P=0.020) (Table 6).

### The first main theme: relevance

Through the quantitative analysis, the Fellowship Program was revealed to be strongly aligned with Tanzania’s healthcare needs. The thematic analysis categorized relevance into 5 sub-themes: country’s priorities, institution priorities, beneficiaries’ needs, healthcare workers’ priorities, and complementing other projects. These were further grouped under 2 broader categories: institutional relevance, encompassing country’s priorities, institution priorities, and complementing other projects, and individual relevance, which included healthcare workers’ priorities and beneficiaries’ needs.

#### Institutional relevance

The Fellowship Program was closely aligned with national strategic goals, significantly contributing to improving public health services. Participants highlighted the Fellowship Program’s role in empowering professionals to teach and train future practitioners, directly supporting national objectives. One participant stated:

“…our country is focusing on improving health services, and when you empower healthcare professionals to teach students effectively, it directly contributes to national goals.” (Clinical expert-007, Dar es Salaam)

At the institutional level, the Fellowship Program addressed critical skill shortages in healthcare. One participant provided a striking example of how the training filled a critical gap in surgical expertise:

“In 2018, there was only one surgeon who had received proper training in neck surgery. So, I was the second person in Tanzania to undergo training in neck and throat surgery, and affiliation (the affiliation was anonymized) is the country’s referral hospital. Therefore, it was also an opportunity for my affiliation to have someone who had specialized in neck and throat surgery.” (Clinical expert-008, Dar es Salaam)

These examples illustrate the Fellowship Program’s alignment with both national and institutional goals, amplifying its overall relevance.

#### Individual relevance

The Fellowship Program was seen as instrumental in building individual capacity, driven by self-motivation, external encouragement, and skill development. Participants valued the learning experience, particularly in expanding clinical knowledge and gaining exposure to new medical conditions and practices. A participant noted:

“I am grateful for what I learned and experienced...while I didn’t achieve all the goals I set for myself, I did learn about conditions like eclampsia, saw how they perform surgery, and gained valuable insights. I was particularly pleased with the ultrasound training, seeing cases that I hadn’t encountered here.” (Clinical expert-005, Pwani)

Self-motivation was a recurring theme. Participants described proactive efforts to apply their training, such as improving medical equipment management. One biomedical engineer shared:

“Management of medical equipment, ensuring proper planning, record-keeping, and being involved in preparing specifications. That, for me, is still vivid, and I have always pushed myself to keep improving in that area.” (Biomedical engineer-003, Dodoma)
  These qualitative findings across diverse training fields validated the quantitative results, demonstrating the Fellowship Program’s relevance to individual and institutional priorities.

### The second main theme: effectiveness

Survey data indicated that 75% of participants reported significant improvements in job performance. Quantitative analysis further revealed a high correlation between effectiveness and workplace application (ρ=0.746, P<0.001). Effectiveness was subdivided into 2 primary sub-themes: training organization, and participant selection.

#### Training organization

The Fellowship Program was seen as well-organized, with many participants appreciating its global exchange of knowledge:

“The distinctiveness of this project is that there were presentations where each country shared its practices. You learn from Ethiopia, Singapore, Congo, and other nations. This sharing of experiences is more extensive compared to projects with local seminars where the learning is more one-sided - receiving information without much sharing of experiences.” (Health Policy and Administration-001, Pwani)

However, limitations were noted. Some participants expressed dissatisfaction with the lack of practical training and language barriers. A medical doctor stated that:

“There was also a suggestion for those we were with; one was a pediatrician, and the other was in surgery. Find a way for them to get hands-on experience because, in medicine, for example, in surgery, it’s hands-on. You can’t learn surgery by watching videos or seeing someone else do it. I suggested they either see how those people practice it or take programs that don’t require physical contact with people.” (Clinical expert-012, Dar es Salaam)

#### Participant selection

The Fellowship Program’s effectiveness stemmed from targeting suitable participants, tailored content, inclusive recruitment, and meticulous organization, ensuring transparency, gender balance, and strong communication by health authorities. One participant highlighted:

“…dealing with maternal health, I wanted to learn about breast imaging, which is beneficial for breastfeeding mothers who may develop abscesses. So, they found a hospital for me where I could learn both.” (Clinical expert-001, Pwani)

Participants commended the gender balance in recruitment:

“When we went, we were 8 in total, and we had a balanced gender distribution - 4 women and 4 men.” (Clinical expert-010, Dar es Salaam)
“I don’t think there is gender bias…. I haven’t seen any gender bias.” (Health Policy and Administration-001, Dodoma)

### The third main theme: efficiency

The efficiency was highlighted in terms of its administrative aspect, which included the terms of management accountability, activities timeliness, and project convergence and financial aspect covering resource allocation. In particular, the training of trainers model was also acknowledged for its training benefits.

#### Administrative efficiency

Participants appreciated the fairness and transparency in the application process:

“Personally, I believe it targeted the right individuals. They assessed who should go, why they should go, and based on what criteria…there was no bias; they looked at qualifications and also considered those who hadn’t received previous training.” (Biomedical engineer-005, Dar es Salaam)

#### Financial efficiency

Participants noted timely financial support and the availability of training materials:

“The availability of materials was good because we were sent them in advance through our emails. They also printed hard copies, so we had both soft and hard copies.” (Health Policy and Administration-005, Dodoma)

Despite its efficiency, many participants suggested extending the training duration for better knowledge absorption.

### The fourth main theme: impact

Survey results showed that 80% of respondents believed the training positively influenced their ability to deliver better healthcare services. This finding was reinforced by a strong correlation between training effectiveness and its impact on job performance (ρ=0.746, P<0.001), as well as participants’ testimony.

#### Individual impact

Participants highlighted tangible improvements in their skills, confidence, and work ethics, which enhanced their ability to handle diverse healthcare challenges. For example, one participant remarked:

“The knowledge and skills we have got are useful in the course of executing our duties. Some have learned laboratory procedures and surgeries, and now they are capable of utilizing such knowledge and skills.” (Clinical expert-001, Dar es Salaam)

The Fellowship Program also facilitated career advancement and academic achievements, including the pursuit of higher education:

“I was a regular nurse, but upon my return, I continued working in the emergency department, eventually advancing to a leadership role.” (Clinical expert-015, Dar es Salaam)
“I even had the opportunity to publish my research paper….yes, I did, during my master’s course. I published it in an international journal with the support of those professors, back in their country.” (Clinical expert-004, Dodoma)

#### Institutional impact

Institutional impacts included improved medical infrastructure, better healthcare quality, and enhanced knowledge transfer. Participants noted that their training contributed to reducing patient referrals and improving healthcare outcomes:

“This has reduced referrals for patients but also assisted the citizens of Tanzania in treating them and providing proper medical care, reducing the burden on the country in terms of diseases and medical costs.” (Clinical expert-008, Dar es Salaam)

### The fifth main theme: sustainability

While many participants felt encouraged to share their knowledge and skills, a lack of formal post-training support hindered the Fellowship Program’s long-term sustainability. Sustainability was categorized into 3 sub-themes: sustainability elements, sustainability strategy, and sustainability barriers.

#### Sustainability elements

Alumni groups formed after the Fellowship Program played a pivotal role in sustaining its impact. Participants highlighted their efforts to provide local training and mentorship:

“We have annual meetings, ongoing activities, and projects for beneficiaries. Alumni groups were formed, and we extend our support to various regions by offering training and assistance.” (Clinical expert-015, Dar es Salaam)
“When I returned, I conducted training and taught all my colleagues. I believe that if they decide to stop supporting, the impact of the program should continue.” (Biomedical engineer-003, Dodoma)

#### Sustainability strategy

Participants suggested increasing the number of trainees and expanding regional coverage to improve the Fellowship Program’s sustainability. They also emphasized the importance of continuing education:

“Most program beneficiaries disproportionately concentrated on Dar es Salaam, Dodoma, and Pwani. Expanding to other regions is essential.” (Clinical expert-013, Dar es Salaam)

#### Sustainability barriers

Key barriers included high staff turnover and financial constraints. Many participants noted that after returning from training, they were transferred to new roles, limiting their ability to apply what they had learned. One participant explained:

“Those who returned to the mother organizations were frequently transferred to new positions or transitioned to new areas.” (Health Policy and Administration-005, Dodoma)

Financial constraints also emerged as a challenge, with participants expressing a need for more sustainable funding to support alumni activities:

“Trainees returned passionately, but no sustainable funding source or platform system was available.” (Biomedical engineer-005, Dar es Salaam)

#### Dissatisfactory outcomes

While many participants expressed satisfaction with the Fellowship Program, some raised concerns about delivery methods, language barriers, and the duration. One participant recommended conducting training within local healthcare facilities to benefit more people:

“Instead of sending one person to Korea for 6 months, bring experts to teach here and train more people simultaneously.” (Clinical expert-001, Pwani)

Language barriers also posed challenges for non-English-speaking participants:

“The translator wasn’t from the medical field, so it was difficult sometimes. But visually, it was easy to follow.” (Clinical expert-010, Dar es Salaam)

Finally, the short duration of training courses limited participants’ ability to absorb the extensive material:

“For me, the time was too short to gain everything I needed.” (Health Policy and Administration-001, Dodoma)

## Discussion

### Evaluating program design, relevance, and inclusiveness through non-parametric analysis

Due to the data’s nature, non-parametric tests including the Spearman ranked-order correlation, Mann-Whitney U test, and Kruskal-Wallis test were employed. Spearman ranked-order correlation coefficients were used to examine associations among evaluation criteria, revealing positive, strong interrelations. The Mann-Whitney U test was utilized to compare independent variables such as gender and employment status, while the Kruskal-Wallis test evaluated differences in medians across groups. These analyses provided robust insights into the program’s long-term outcomes despite limitations in using parametric methods for Likert-scale data.

As shown in Table 5, the Spearman ranked-order correlation coefficient results confirmed that the program was well-designed, as it indicated a positive association across the Fellowship Program’s evaluation criteria. This implies that the program effectively reflected the needs and priorities of the health sector in the country. Such alignment can be attributed to KOFIH’s close collaboration with health sector partners, which enabled the Fellowship Program to continuously assess and incorporate the training needs of participants. Regular demand surveys conducted in partner countries further supported this alignment by identifying specific needs and priorities.

Additionally, while the Kruskal-Wallis test (Table 6) demonstrated a significant difference in perceived training content relevance across age groups (P=0.020), other results confirmed the program’s relevance regardless of participants’ age, type of course attended, or the institution they were affiliated with before training. This indicates that the program is largely inclusive. Although the Fellowship Program has primarily targeted clinical experts, health administrators, high-level officials, biomedical engineers, and infectious disease specialists, it has also promoted an enabling environment that could accommodate other cadres of the health workforce across the country.

### Contribution to strengthening the health system through tailored approaches

This study examined the Fellowship Program, targeting a broad spectrum of health workers in Tanzania, including auxiliary staff, in contrast to the more commonly reported focus on rural medical professionals or doctors [6-8]. The findings highlighted the importance of aligning training initiatives with local healthcare contexts and working conditions. The relevance was evident in its alignment with Tanzania’s specific health sector needs; this factor was strongly associated with participant satisfaction and role-specific relevance, as noted in previous studies [9,10]. By addressing the unique challenges faced by health professionals, the Fellowship Program demonstrated a high level of efficacy and optimal use of resources. Success was particularly attributed to the delivery of targeted educational modules and learning strategies tailored to local demands [9].

### Minor dissatisfaction, but major considerations

While the Fellowship Program had a significant impact, some limitations warrant attention. The study revealed questions about whether the training sufficiently reflected the local Tanzanian context and work conditions. A participant (Clinical expert-002, Dar es Salaam) noted, “participants acquire a lot of knowledge; however, when participants return home, things become impractical.” Hands-on learning was highlighted as a notable gap, particularly in clinical courses [11-13], which diminished the Fellowship Program’s long-term effectiveness and sustainability. Additionally, the poor visibility of KOFIH and weak integration with bilateral projects were pointed out as areas needing improvement by the participants. Insights from participants emphasized the importance of addressing these gaps to enhance training quality.

### Interpretation

This study provides groundbreaking insights through a mixed-methods approach, offering valuable national evidence on the Fellowship Program. In the OECD-DAC framework, the implementation of the Fellowship Program is in line with each criterion to strengthen Tanznia health systems. In particular, the results show the Fellowship Program’s strong alignment with Tanzania’s health priorities, effectively addressing skill gaps through tailored training modules. High relevance and effectiveness scores demonstrate the Fellowship Program’s success in meeting professional needs, boosting participants’ confidence, and enhancing institutional service quality. However, lower sustainability scores reveal challenges, including limited follow-up support, insufficient practical training, and disconnects between training conditions in the host and home countries. These findings emphasize the need for contextualized learning approaches, better integration with local systems, and improved post-training support to maximize the long-term impact and sustainability in strengthening Tanzania’s health workforce.

### Comparison with previous studies

The findings of this study align with the short-term effects reported in similar programs [3,4,14]. However, the evaluation of long-term sustainability—an area less explored in the existing literature—adds to the originality of this research. In this context, comparing the study to previous studies highlights the need for a focused analysis on sustainability [3,4,6,14]. Notably, the low score for sustainability (mean=58.01) appears to be associated with the high turnover rates of alumni and the lack of post-program support. To address these challenges, we propose extending local training initiatives and enhancing alumni network activities as part of post-program management. Current efforts to strengthen alumni networks through KOFIH Global Alumni should be expanded through diverse strategies, with the aim of disseminating these approaches within the host country.

### Limitations

This study offers groundbreaking insights into the effectiveness of the Fellowship Program through a mixed-methods approach, providing valuable national evidence. However, 2 significant limitations must be noted.

First, among the 140 alumni, 97 participated in the online survey, and 30 engaged in IDIs, with 6 of these participants also involved in the FGD. While the response rate is commendable, given the challenges of tracking first-batches of participants, the study is limited by sampling bias. Specifically, there is a regional bias as the sample is restricted to Dar es Salaam, Dodoma, and Pwani regions, and an overrepresentation of alumni from specific years (2016, 2021, and 2022). Additionally, the omission of participants from the Infectious Disease Control and High-Level Official courses might further limit the study’s representativeness.

Second, the efficiency evaluation method poses limitations. Efficiency, as defined by the OECD-DAC, assesses how resources are utilized and whether the intervention achieves results economically and in a timely manner [5]. However, this parameter could not be comprehensively evaluated due to the unavailability of essential data, such as the basic unit cost (i.e., the cost per individual for one-time participation in the Fellowship Program). These methodological constraints prevented a comprehensive assessment of resource input and efficiency.

These limitations underscore the need for a cautious interpretation of the results and highlight the necessity of further research with a broader sample and diverse settings to validate and expand upon these findings.

### Suggestions: possible alternative models for better outcomes

The study proposes 2 alternative approaches: (1) deploying trainers to local settings to provide on-site instruction and (2) adopting distance learning modalities such as e-learning platforms and online courses [6]. While distance learning is limited for clinical training that requires hands-on practice, it offers increased accessibility, reduced workplace disruptions, and equitable learning environments. Integrating these approaches could enhance training flexibility and scalability while addressing current limitations.

### Conclusion

The Fellowship Program was modeled after the Minnesota Project, which significantly impacted health systems, medical research, and medical education in Korea following the Korean War through the American Development Program [15]. While the success of this project in the Korean context is noteworthy, it may not be directly transferable to other settings due to unique historical and cultural factors. These contextual differences necessitate careful consideration when generalizing findings to diverse environments.

Nonetheless, this study highlights the positive impact of the Fellowship Program on healthcare delivery in Tanzania. The program has demonstrably enhanced the skills, knowledge, and service quality of Tanzania’s health workforce. Its alignment with local health sector needs has strengthened technical competencies, boosted confidence, and supported professional development, ultimately contributing to improved care delivery and capacity-building initiatives.

However, gaps in delivery methods, contextual alignment, and practical training highlight areas for improvement. In particular, there is substantial room for improvement in sustainability, and strengthening this gap will produce different results in comparison to previous studies. To do it, adopting strategies such as specialized personnel deployment and distance learning could extend the Fellowship Program’s reach and sustainability. At the same time, with continued evaluation and refinement, the Fellowship Program can further support Tanzania’s health system and drive long-term, meaningful advancements in service delivery.

## Figures and Tables

**Figure f3-jeehp-22-06:**
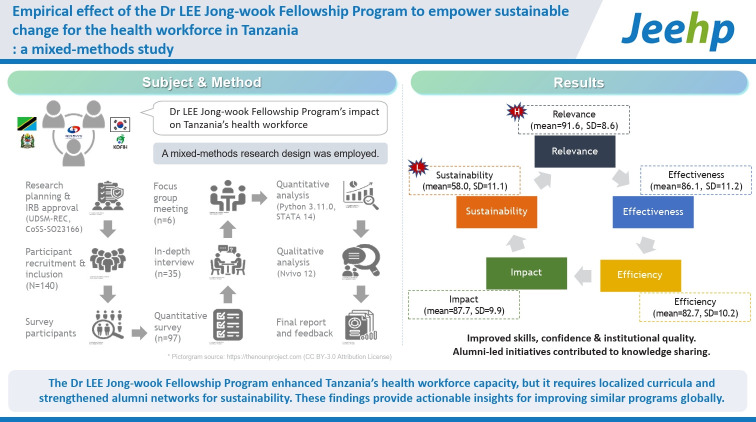


**Table 1.** Specific questions for each criterion (relevance, effectiveness, efficiency, impact, and sustainability)^a)^

Criteria	Question	Definition
Relevance	Is the intervention doing the right thing?	Whether the intervention objectives and design correspond to the beneficiaries’, country’s, and partners’ needs, policies, and priorities.
Effectiveness	Is the intervention achieving its objectives?	Whether the intervention achieved, or is expected to achieve, its objectives, and its results, including any differential results across groups.
Efficiency	How well are resources being used?	Whether the intervention delivers, or is likely to deliver, results in an economic and timely way.
Impact	What difference does the intervention make?	Whether the intervention has generated or is expected to generate significant positive or negative, intended or unintended, higher-level effects.
Sustainability	Will the benefits last?	Whether the net benefits of the intervention continue, or are likely to continue.

a) The question and definition were adopted from the OECD-DAC [5].

**Table 2.** Reliability test results (Cronbach’s α)

Evaluation criteria	Items	Cronbach’s α value
Relevance	5	0.675
Effectiveness	7	0.895
Efficiency	7	0.687
Impact	10	0.897
Sustainability	3	0.779

**Table 3.** Descriptive characteristics of the respondents

Characteristic	Quantitative data (%)	Qualitative data (%)
No. of respondents	97	35
Region		
Dar es Salaam	61 (62.9)	17 (48.6)
Dodoma	12 (12.4)	8 (22.9)
Pwani	10 (10.3)	10 (28.5)
Kilimanjaro	5 (5.2)	
Mbeya	4 (4.1)	
Songwe	1 (1.0)	
Iringa	1 (1.0)	
Manyara	1 (1.0)	
Singida	1 (1.0)	
Kisumu (Kenya)	1 (1.0)	
Gender		
Male	55 (56.7)	21 (60.0)
Female	42 (43.3)	14 (40.0)
Age (yr)		
25–34	21 (21.6)	
35–44	42 (43.3)	
45–54	30 (30.9)	
55–64	2 (2.1)	
≥65	2 (2.1)	
Institution		
Ministry of Health	12 (12.4)	3 (8.6)
National hospitals	35 (36.1)	15 (42.9)
National lab	11 (11.3)	
Specialized hospital	4 (4.1)	
Zonal referral hospital	6 (6.2)	4 (11.4)
Regional referral hospital	10 (10.3)	9 (25.7)
District hospital	2 (2.1)	1 (2.9)
Higher learning institute	9 (9.2)	3 (8.6)
NGO or private sector	8 (8.3)	
Training courses		
Clinical courses	49 (50.5)	24 (68.6)
Biomedical engineering	21 (21.6)	3 (8.6)
Health policy and administration	13 (13.4)	3 (8.6)
Infectious disease control	12 (12.4)	
High level official course	2 (2.1)	
Not applicable^a)^		5 (14.3)
Year of training		
2009	3 (3.0)	2 (5.7)
2010	5 (5.2)	4 (11.4)
2011	2 (2.0)	3 (8.6)
2012	6 (6.2)	2 (5.7)
2013	6 (6.2)	3 (8.6)
2014	8 (8.2)	3 (8.6)
2015	5 (5.2)	1 (2.9)
2016	10 (10.3)	2 (5.7)
2017	6 (6.2)	1 (2.9)
2018	5 (5.2)	2 (5.7)
2019	6 (6.2)	2 (5.7)
2020	5 (5.2)	1 (2.9)
2021	10 (10.3)	2 (5.7)
2022	20 (20.6)	2 (5.7)
Not applicable^a)^		5 (14.3)

NGO, non-governmental organization.

a) Five individuals were non-participants of the Fellowship Program (i.e., health sector leaders).

**Table 4.** Descriptive statistics for evaluation criteria

Evaluation criteria	Mean±SD	Min	Max
Relevance	91.6±8.6	65	100
Effectiveness	86.1±11.2	57	100
Efficiency	82.7±10.2	20	100
Impact	87.7±9.9	54	100
Sustainability	58.0±11.1	20	73

SD, standard deviation.

**Table 5.** Spearman rank-order correlation coefficients of the Fellowship Program’s evaluation criteria

Variable	Relevance		Effectiveness		Efficiency		Impact		Sustainability	
	rho	P-value	rho	P-value	rho	P-value	rho	P-value	rho	P-value
Relevance	1									
Effectiveness	0.688	0.000	1							
Efficiency	0.294	0.004	0.400	0.000	1					
Impact	0.602	0.000	0.746	0.000	0.505	0.000	1			
Sustainability	0.417	0.000	0.538	0.000	0.411	0.000	0.587	0.000	1	

**Table 6.** Kruskal-Wallis test of the Fellowship Program’s relevance across age, type of courses, and institutions

Relevance statements	Age of beneficiary		Type of training course		Type of institution	
	Kruskall-Wallis	P-value	Kruskall-Wallis	P-value	Kruskall-Wallis	P-value
Training content beneficial to work	11.606	0.020^*^	5.759	0.217	0.194	0.978
Overall training beneficial to work	3.969	0.410	1.227	0.873	0.077	0.994
Facilitator helpful during training	2.855	0.582	4.501	0.342	4.163	0.244
Training relevant to country needs	5.576	0.233	2.693	0.610	1.096	0.777
Training relevant to work priorities	2.881	0.577	1.564	0.815	3.646	0.302

* P<0.05.

## Footnotes

**Data availability**
Data files are available from Harvard Dataverse: https://doi.org/10.7910/DVN/MBK4CC Dataset 1. Qualitative research dataset comprising transcripts of in-depth interviews and the focus group discussion.

Dataset 2. Cleaned quantitative research dataset.

## Supplementary materials

Supplementary files are available from Harvard Dataverse: https://doi.org/10.7910/DVN/MBK4CC

Supplement 1. Quantitative and qualitative questionnaires.

Supplement 2. The cumulative number of participants in the Fellowship Program.

Supplement 3. Audio recording of the abstract.
