# Supplementary material for: Empirical effect of the Dr LEE Jong-wook Fellowship Program to empower sustainable change for the health workforce in Tanzania: a mixed-methods study
Source: J Educ Eval Health Prof. 2025 Jan 20;22:6. doi: 10.3352/jeehp.2025.22.6 (PMC12003955; doi:10.3352/jeehp.2025.22.6)
Supplement: Supplementary file 1 [file jeehp-22-6-dataset1.docx]

**KII WITH CLINICAL EXPERT -TUMBI, PWANI**

## **PART 1: INTRODUCTION**

| **Date** | 20/11/2023 |
| --- | --- |
| **Region** | PWANI |
| **Location** | TUMBI |
| **Language used** | Swahili |
| **Translated** | English |
| **Gender** | MALE |
| **Position** | Critical care expert |

**I: Okay, let’s go straight to our questions, and perhaps first of all, Doctor, I would like to know from you how this project, which you had the opportunity to go to Korea, aimed to address the priorities of healthcare providers in this region, please**.

P: Yes, because the goals of KOFIH were in mother and child health, specifically targeting that area. They were not focusing on any other side, so in our Tumbi hospital in Kibaha, we were really focused because it was necessary to implement those goals within that framework. There was a building there for mother-child health, and they dedicated themselves to providing funds to make that building specifically for maternal and child health services. It was a grade one building, and the floor above was grade two, and below was the children’s ward. We agreed with the hospital management and partners in Kibaha that the upper floor, which was grade one, should be special for pregnant women who have undergone surgery or are waiting, and there was a separate area for health insurance. Here, the building looks like you’re going to the ground floor when you come from above, but it was actually counted as the first floor because the children’s ward was in the basement. So here, mothers who had given birth were staying, and on the other side, a building was constructed that matched the theater specifically for mothers. So pregnant women and those with other gynecological problems were served here, and there was also a radiology department below. Of course, you can’t say this is for mothers and children only; it received services for all hospital patients.

Then, we improved the children’s ward, divided it into sections for children of various ages, and created an emergency area for receiving children in critical condition. On the far side, we created something called a pediatric ICU, that is, services for children who are overwhelmed, aged 0-5 years, along with a Neonatal ICU for those aged 0 to 28 days. Those preterm babies had their special department, you know, when taking care of preterm babies, many precautions need to be taken to prevent infections; otherwise, if preterm babies get infected, it becomes a problem. So, we succeeded a lot with the help of KOFIH; they provided delivery kits and abdominal services to mothers. You find one package, but it contains all the equipment for a complete abdominal operation. The equipment was really impressive; even the former president of the fourth phase, Honorable Jakaya Mrisho Kikwete, came to inaugurate it. He was pleased to see that the equipment was complete. Despite focusing on maternal and child health services, they also provided a large generator they call Caterpillar, near the water tank. It was automatic; for example, when the power drops to 220 volts, it automatically starts. So, it solved the energy problem, especially in this building for mothers and children. Additionally, I was able to convince them to build an incinerator specifically for medical waste.

**I: Those KOFIH people!?**

P: Yeah, KOFIH built us a little; there were many questions, a very long discussion, but I was able to convince them because even when I went to Korea, they asked me why we wanted them to build an incinerator!? I said you guys have focused on maternal and child health services, so these mothers who come to give birth, once they finish giving birth, they remove the placenta, and it is collected somewhere. Now we usually take it and burn it in a special place. So why not have a facility that is acceptable and approved by the health ministries? I don’t think you would be pleased if we were here talking, and you see a dog or a vulture flying into a bucket, and it comes out with placenta; you wouldn’t be happy at all. They understood me, and they built a very modern incinerator. It was built by a person named Professor Manyere; I think you know him!?

**I: Mmmh yeah!**

P: He was the head of the Engineering Department at the university, and later he was appointed as the Chief Government Chemist until he retired. He was the one who built this, and he had built many other places, even outside the country.

**I: Okay, as we know a country may have its own priorities. I wanted to know if the project was in line with the country’s priorities or not! Especially in the health sector!?**

P: Yeah, they were in line because priorities, for example, in the health sector, must align with those of the WHO. At that time, we had something called MDG 5; I don’t know if you’ve heard of it!?

**I: Yeah**

P: Millennium Development Growth 5, so those are the ones we were targeting. KOFIH was also targeting them, and they were included in the Ministry of Health’s package. So, we were in line with KOFIH. You can go to an NGO in Southern Korea, but it is overseen a lot as a government; they can’t do anything without the government knowing because the Korean aid organization is significant, called KOIKA. But KOFIH can’t do anything without KOIKA knowing, and KOIKA is the government, so everything was well-known. Nothing was done outside the priorities of the government. When KOFIH went to developing countries, they had a joint venture with the lottery clubs in those countries. In Korea, they had a joint venture with the lottery clubs. So here, they had to negotiate with the lottery club, and they were working together during that time. I remember Mr. Chande used to come a lot to oversee the project because it was a must. Even the Lottery Club had its percentage; they financed. So, it was something well-known. The late Saendi Chande, the people who spoke to the heads of state were direct, so it was well-known. It had 100 percent government backup.

**I: 100 percent!?**

P: Yeah

**I: Perhaps, another thing I wanted to know is that, there might be other projects being implemented in Kibaha apart from Dr JLW project. Now, do you think this project aligns with other projects being implemented in your area/ region!?**

P: Ah, align in the sense of that aspect or!?

**I: Yeah, in terms of its priorities, and may be sort of activities of the project implemented. Do you see them aligning with other projects?. Were they similar to those of other projects? Yes please!.**

P: Priorities must align. That’s why, even in the Ministry of Health, when we started, there was communication between them and us at Tumbi Special Hospital, which was under… but… the hospital center, I can say, is under TAMISEMI. Still, in health activities, it is directly overseen by the Minister of Health. So, in any case, they must align; they can’t be peculiar, they must be unique and fit. It must be that you have taken something from Korea, and it fits, and you get recognition. For example, if the goals of the ministry say to reduce infant mortality rate, which is death below one year, now, those priorities must align, reduce under-five mortality rate; they must align, reduce prenatal mortality, meaning death below 28 days; they must align, reduce maternal deaths; they must align. So, those are their priorities, but they align with the goals of the Ministry of Health. The Ministry of Health extracts them from the national vision, for example.

**I: Now I would like us to look at the health committees present at the level of the region, district and even health facilities. At the regional level we have RHMT, at the level of district we have CHMT and other health committees at the level of facilities. At all these levels, the aforementioned committees may have their priorities which are in line with national priorities. My aim is to understand how this project aligned to priorities of different health committees in those levels. Please!**

P: Yes, so when they came to Special Hospital, which was under the KIBAHA EDUCATION CENTER but the government had appointed that hospital, even though it was under the organization, Kibaha was supposed to be the regional referral hospital for the Coastal region. Despite the peculiar geography of the Coastal region, as you know, it’s quite unique. There is the coast in the south, when you go to Morogoro, there is the coast. In Bagamoyo, there’s the coast. So, there’s Kisarawe, Mkuranga, and Mafia across the Indian Ocean. In Kibiti, you can’t say a patient couldn’t be managed in Kibaha or Bagamoyo; they should be brought here to Tumbi. If it’s not possible, then they go to Muhimbili. No, they are taken directly to Mkuranga or Muhimbili. Even though after providing these hospitals, Amana, and Temeke, they are counted as regional hospitals, Mkuranga can’t send someone; however, cases from Temeke can’t go to Muhimbili; they go to Amana. You see! Bagamoyo doesn’t send to Mwananyamala; they go directly. Now, we, as a regional hospital located in the Kibaha district, face challenges due to the coastal geography. Kibaha district has two councils: Kibaha town council and Kibaha district council, you see? Even though it’s one district, the administrations are different. So, even though I was the Medical Incharge at Tumbi Special Hospital, the Regional Medical Officer (RMO) oversaw me. I was responsible to the RMO, and at the same time, I was also responsible for the Kibaha administration. When KOFIH came, before they left, they had to get feedback from the Regional Health Management Team (RHMT). They used to conduct ABCD activities and presentations. So, the RMO and his RHMT knew what was happening. RHMT is a formal amalgamation in the health sector. It receives guidelines and messages from the Ministry of Health. The instructions come from the Ministry to the Regional Medical Officer and the RHMT, then to the Council Health Management Team (CHMT). So, that guidance doesn’t bypass anyone. Even though I was in charge of Tumbi Special Hospital, I couldn’t be bypassed because, after all, we are governed by these guidelines, WHO committee, Ministry of Health, RHMT, and everything.

**I: Now, I was wondering if you could tell me the entire process of the project implementation. Particularly, your thoughts on how participants were obtained. Can you tell me how participants/ beneficiaries of this project were selected please?**

P: Are you asking about all the participants or just me? (Laughs)

**I: You can tell me how you were selected and may be others if you have any idea please. Did you go through the interview or what exactly?**

P: Because this KOFIH project in Tanzania started at Tumbi Hospital, Kibaha Clinic and Services were privileged. Initially, when the project team came, those people didn’t even know English. They had prepared to go to Central African countries, make a round, and select one. However, when they arrived here, they were impressed with our hospitality. I, coming from the hospital with my director, who was very impressed, had discussions with them in the boardroom over a weekend. He called the top leaders, and many stakeholders came. We discussed and communicated through email. They informed us that they chose Tumbi Special Hospital to start the collaboration for Dr. Lee Jong-woo’s project. They said a team would come, and when Mr. Jan and others came, we had discussions. After they signed and confirmed their interest in mother and child health, I, being on the hospital’s leadership side, was appointed as the Health Services Manager and Medical Officer in charge of the hospital. We established a common language. They issued an invitation with tickets, and we went for three months in 2009. We met people from other countries, had an orientation week, and went through training in our respective departments. Towards the end, we gathered at the KOFIH residence hotel where we were accommodated. We suggested that three months were too short, especially considering the living conditions, and they extended it to six months. Some, looking at it, proposed one year. After our return, the next invitations specified the need for certain professions, and the hospital management convened to decide who was suitable.

**I: You can talk a little bit about your intake. Were you only male or there were also female?**

P: Yes, only male doctors.

**I: Oh, you said three?**

P: Yes, three. Later, when they came again, they said they couldn’t involve hospitals from Dar es Salaam City Council. I suggested it might be possible, so we left with them. We went to the Chief Medical Officer of Dar es Salaam City, signed the agreement, and they suggested positions at Temeke, Amana, and Tumbi. Of course, they left with three, and later, they proposed, “Can’t we involve Muhimbili?” They said, “MUHAS, then.” So, I told them that if we’re involving a higher training institution in health training, we need to go to the Ministry and see the Secretary-General, present the idea. We went to the Ministry, and after introducing them, they agreed. They were already waiting for them since I had called. We discussed it thoroughly, and then they started taking them. It continued to extend to Dodoma and beyond. There were no interviews, but they specified the profession, and the administration chose based on their needs. For instance, I once made a special request. Since Tumbi Special Hospital, being on the Morogoro Highway, handles 7% of motor transport accident victims in the United Republic of Tanzania, I requested someone from the Emergency Medical Department to get training. I suggested a one-year training, and they accepted. I sent a doctor for a year, and when he returned, he greatly assisted. Many requests came in, and some went for a whole year, taking advantage and studying.

**I: So, the project seemed to have achieved them, right?**

P: Oh, truly, they were achieved. Because the maternal and child health services provided at Tumbi Hospital are, I can say, different. I personally took a message to the staff, more than 30 of them, including nurses and doctors. When they returned, nurses usually had a plan for rotation, but, for example, if they went to the guiding side, they wouldn’t be moved, although there was a gap after Mloganzila Hospital was established. KOFIH wanted all those who had undergone training with Dr. Lee Jong-woo in South Korea to be the founders there. The majority went, and those who remained were only two, Linda and Lila. But the majority are at Mloganzila. Some, later, developed an interest. For example, Dr. Daudi Ntunambuzi, who was called CMT, later did a Master’s, went to take a Master’s there, and when he went, he stayed at Muhimbili.

**I: Alright.**

P: So, everyone had their luck, but the goals were truly achieved. They did a great job, as people say these days, they should be given their flowers.

**I: ‘‘They should be given their flowers’’, hahahaa!. Okay,iIf that the case then, what if I were to ask you, what it meant exactly for you being part of this project. What if you had to miss such opportunity of being part of this project, would things be different? or would you regret missing it?**

P: Ah, in short, because I was born during the era of Ujamaa and Self-Reliance politics under the late Mwalimu Nyerere, the guiding book said, “Position is a responsibility, and the education I receive is for the benefit of the public.”

**I: Yes.**

P: So, I was educated, and I can’t boast that I was educated by my siblings or with taxpayers’ money because now various institutions in the country have initiated a system called Community Social Responsibility, meaning sustaining the community. So, I don’t see at all that I lost anything by going there because the education I received is to benefit the country and the citizens. Especially for me, being a Pediatrician, a specialist in children’s diseases, every child is valuable to me, even if they are not mine, they have great value. So, if I see a child suffering and in pain, instead of just taking a photo or chatting, I will ensure their well-being. I fight for it. I ensure their lives. So, for me, going there was an opportunity. The extra knowledge I gained there, I am obliged to deliver it to the community.

**I: Ah, okay, good that you brought me to a point I just wanted to kind of,..ahh.. to look at. Let’s start right there with the point you ended with. what would you say if you are asked to say a little bit about the how the project has benefited you individually and the institution you were working for? Starting with you individually, then we will look at the level of institution.**

P: It benefited us in that, first of all, Koreans, in their history, if you had the chance to go there, they used to amuse us, saying something like, “In the countries you came from, and especially referring to Tanzania, right now, and we also went through that stage, we fought, and fought, and even for the health insurance, we didn’t have health insurance for everyone. But we started from there, gradually, and gradually reached here. We have built a strong work ethic, as you also hear in the media. Here, once someone is caught in corruption, we dismiss them. So, their comparing themselves to us, 40 years ago, saying they were like us, and now they are where they are, we were amazed. But the effort they put into their work, we thought they deserved it because I’ll give you an example Because on the first day after finishing that orientation week, we were taken to the hospitals where we would undergo our training. On the first day I arrived there, I was assigned to a resident. There were residents, and there were others on the path to becoming associate professors. Now, the resident I was assigned to, after completing rounds, imagine, here at work when you report, some people show up at half-past one, others come at two, I don’t know what time. We sit down to compile reports, not sure when we’ll finish. The report time here is undefined. In Korea, you walk towards the hospital, and cars are rushing, professors heading there. At exactly 12, the ward round begins. So, when I explained to the resident that tomorrow’s reporting time, what time should it be? He laughed and said, “That’s a very good question. If the professor starts the ward round at 6 am, what time do you expect to be here?” I told him it should be before 6 am. He said, “Very good, so you have understood.” The discipline of our colleagues there, it’s like a trick; the professor is already there at quarter to 12, by 5 minutes to 12, he’s in the ward, he’s wearing his white coat. You start rounds, and by around one and a quarter, one and a half hours, you’ve finished the ward round for all the rooms. He informs about all the sick children, leaves, goes to his office, gets a cup of coffee. They don’t care; you can do rounds while having your cup of coffee. They have those cups with lids, very simple. So, what I want to say is that one of the things I gained in terms of skills after going there is work discipline, time consciousness, and, of course, respect for those above you and love for the patients you are serving. Those are the fundamental things there. They serve any patient without caring who the child belongs to, whose child this is. So, there were some special cases; for instance, there were twins born, their father was a specialist doctor. They were treated just like the others. Now, when I returned, our director at that time

You’re being asked specific questions, so I did a presentation and was asked many questions. I was asked, “Are these things you’ve learned there applicable here?” So, I made it clear that I would be able to implement what I learned there. Apart from the presentation at the management center, I also made a presentation to the hospital management team and the Tumbi Special offers staff in general. This was so that even those who didn’t have the opportunity to go there would understand and be informed about the systems we learned in South Korea. They are not very different from our Japanese counterparts. They don’t have a hierarchical structure like having individual offices for managers. It’s more like a cafeteria. If the boss is there, he sees everyone when they leave, and he knows if you were absent and why. I do this to maintain discipline and ensure collective responsibility is high.

**I: Were you provided with an allowance during training ?**

P: Yes, we were given an allowance.

**I: Was it given on time?**

P: It was given on time. They had a system where we were paid weekly. When you went there during the orientation week, you had to be processed. You opened an account at their bank, and every week the money would enter on time.

**I: In general, you’ve explained how the project helped you in terms of building your capacity. I wanted to know, if you are to compare your past experience before attending the training and after. Did you notice any difference or was almost the same as those who did not attend?**

P: Ah! I once got a presentation during Jayka’s training titled “Change the World.” If you don’t accept change, then you are not human. So, I had to change because change is mainly based on three things: knowledge, attitude, and practice. Knowledge is what you have in your head, attitude is how you manifest in the community, and practice is how you manifest your work with patients based on… so, this attitude was more on the professional side because I had to show that I had changed. This included aspects like time consensus, effectiveness, cost-effectiveness in financial usage, for example, and time management. And other things like when you’re a leader, you need to know how to motivate people. Motivation is not always monetary; you can verbally speak to an employee, and they leave satisfied even if they came with pressing issues. So, these were the changes I made when I returned.

**I: As we are finishing, there is one thing I want us to look at. For projects such as this, many of us would want to see sustainability of the project outcomes and activities. All we want to see is that even when the funding ends, still the activities and outcomes should be there. In your view, do you see it coming in this project?**

P: Yes, the project’s progress cannot stop because KOFIH’s goals were not limited to them. Even though their main interest was in mother and child health, like one day, I watched TBC1, and there was a doctor explaining or it was a news report. We went to New Africa, where they have a machine for diagnosing various issues in a baby while still in the mother’s womb. This can help in gynecology, and if any issues are detected, they can be addressed. The important thing is that they have a joint system. Their interest is in mother-to-child health, but mother and child health is not only their concern. WHO broadened the scope, including RHMT and CHMT, so it won’t stop. There’s an evaluation done every five years, both at their level and at the ministry and regional health management team levels. So, I don’t expect that it will reach a point where they will get stuck. I see the project as having a wide impact and spreading across the country, incorporating various hospitals.

**I: Just to conclude, what do you think need to be prioritized to improve and ensure that the project achieves its target more and more ?**

P: Well, luckily, during my leadership at Tumbi Special Hospital, many programs came from different angles. NGOs, like Columbia Invest, facilitated by many organizations under USAID, handled various aspects, such as HIV/AIDS treatment. DEICA focused on administration and capacity building for health workers. Many programs have come and gone, reminding us that they won’t be there forever. Therefore, it was essential to absorb the knowledge and, in our group work, document the information to disseminate it to others who didn’t attend. Regarding KOFIH, they initiated the Dr. Lee Jong program, and he wanted every country globally to have excellent health services. Unfortunately, he passed away during his tenure as WHO Director.

**I: You were talking about what should be improved.**

P: To improve, continuous training isn’t the answer. I was emphasizing this during the Corona period, when everything came to a standstill globally. Many organizations reminded us that they wouldn’t be here forever. The important thing now is that the capacity building has been done. What is needed is for us to utilize the knowledge. The basic premise is that we should stop living like nestling birds waiting for the mother to come and chew food and feed us. We need to start using the knowledge that has been highlighted. Others have been using it because they have given challenges to go and do these activities of writing those topics and disseminating information. They continue to provide capacity building. So, they’ve done a lot. It’s time for us to change and deliver what is supposed to be delivered, perform community social responsibility, and show them that people who came for training are now performing wonders. The multiple effects are evident.

**I: Wow! Doctor, I think I’ve got more than enough. So, I thank you so much for your time. It has been very fruitful.**

**KII WITH RADIOLOGIST TUMBI PWANI**

| **Date** | 20/11/2023 |
| --- | --- |
| **Region** | PWANI |
| **Location** | TUMBI |
| **Language used** | Swahili |
| **Translated** | English |
| **Gender** | Male |
| **Position** | Radiologist |

**I: To begin with, I’d like to know from you how Dr. Lee’s project aimed to address the priorities of healthcare providers in this field.**

P: When this project came to Tumbi Hospital, I went there from another hospital… and when the Koreans came there, it was a project focused on mothers and children. As a radiologist, I was involved in Imaging, specifically ultrasound for pregnant women and also for young children. Additionally, for maternal health, it involved the first imaging of breast X-rays for screening breast cancer and more. So, I went there under this umbrella, directly impacting the health of both mothers and children. Here, we target the child after birth, even looking at their health in the womb through ultrasound.

**I: Let’s look at the entire process of getting selected. Could you tell me how did you get the opportunity to be part of the project as one of the selected participants? What process did you go through?**

P: For doctors, two of us went along with three nurses. The doctor who went was a pediatrician. When they sent the invitation, stating they needed someone related to mother and child, we, representing other professions, argued for the inclusion of obstetric ultrasound. Luckily, during that period, I was the only radiologist, so there was no other competing radiologist. They interviewed me over the phone after learning about my profession, asking about my expectations for the project. I expressed my interest in learning obstetric ultrasound in detail to identify pathologies in unborn children. Also, dealing with maternal health, I wanted to learn about breast imaging, which is beneficial for breastfeeding mothers who may develop abscesses. So, they found a hospital for me where I could learn both, and I gained the knowledge in one and a half months, as the overall duration was three months.

**I: Which year did you go?**

P: 2010.

**I: How many participants attended that intake from Tumbi Hospital?**

P: From Tumbi, we were a total of five. Two doctors, a pediatrician, and myself went first. After a month, three nurses joined, specializing in child and labor hood care.

**I: How about the aspect of gender. Were you only women or there were also men ?**

P: In the initial part of the journey, it was mostly women. However, after the first month, given the large number of participants that year, men from the biomedical field joined. There were two or three men, and they were sent later.

**I: So, the biomedical participants joined later?**

P: Yes, they joined later. We started with two doctors, then three nurses came, and finally, the biomedical technicians, who were experts in equipment. The argument was that although the Koreans provided equipment, we needed local technicians for repairs. So, our technicians were selected and trained.

**I: When the project was coming, and you got the opportunity to go, did you see this project as aligning with the country’s priorities, especially in this healthcare sector?**

P: Of course, because it was during the period when we were fighting to reduce maternal and child mortality. They aligned with the Millennium Development Goals, and at that time, the Ministry of Health was actively working to prevent maternal and child deaths. For example, in my field of radiology, performing obstetric ultrasound helps identify early risks for the child, such as the risk of intrauterine death or death during labor. It becomes easier to advise the doctor and suggest, for instance, that the child be delivered prematurely to ensure proper care or that the mother undergoes specific treatment to carry the pregnancy to term safely. So, obstetric ultrasound helps detect various risks for the child while in the womb, including those that could lead to maternal death during childbirth.

**I: Have you ever seen other projects focusing on this healthcare sector before the KOFHI project? Before attending and looking at the doctor, have you observed other health projects, and if so, how does this project align or differ from others in this field?**

P: The distinctiveness of this project is that, while it aims to reduce maternal and child deaths, it focuses more on building the capacity of healthcare workers. In capacity-building projects, you often find local seminars, but in this case, they believed that sending participants abroad would facilitate sharing experiences with other countries. During the first week, there were presentations where each country shared its practices. You learn from Ethiopia, Singapore, Congo, and other nations. This sharing of experiences is more extensive compared to projects with local seminars where the learning is more one-sided—receiving information without much sharing of experiences. Going abroad allows participants to meet people from different countries, expanding the scope of shared experiences and learning from each other.

**I: Alright. At the beginning you tried to explain how the project aligned with the country’s priorities and even the global agenda and plans. Now, when it comes to the structure in the healthcare sector, we have committees such as RHMT at the regional level, CHMT at the district level, and health committees in specific health facilities. These committees may have their priorities which also align with the national priorities. I would like to know if this project aligned with the priorities of these regional, district and health facility health committees.**

P: Well, it aligned, as I mentioned earlier, the goal of the hospital or RHFT/CHFT was the same – to reduce maternal and child mortality. However, in achieving this goal, there are various aspects. The Ministry and its departments may focus on building numerous health centers and sending professionals who may have graduated recently. Even though these professionals have their education, they still need additional skills and the opportunity to share experiences. So, it’s like complementing the national goals. The government establishes health centers, bringing maternal and child services closer to the people. They educate professionals in our Tanzanian institutions, and KOFHI complements this by enhancing their skills and sharing experiences with other countries. As you know, Korea developed not just by progressing but by learning from others. So, when professionals are sent there, they see how others work and bring that knowledge back. Even though individuals may return with a work ethic, they might be discouraged by those who haven’t had such experiences. However, having the attitude and spirit of work is crucial. When you see that others have progressed and are working hard, it motivates you. It’s not just about the techniques; it’s also about the work ethic and spirit. So, sending professionals there changes their attitude positively.

**I: Haha, that they became “Koreanized.”**

P: Haha.

**I: What are your thoughts on the entire process of getting beneficiaries/ participants. Was there transparency in the process of selecting participants/ beneficiaries?**

P: Well, in terms of participation, we were talking to the management about helping them select participants gradually. That’s why in the first, second, and third years, people went gradually. After that, they started expanding to other areas, and the goals of maternal and child health seemed to be fading because participants were going to different fields. However, for us at Tumbi, it was primarily about maternal and child health. Even when we later sent someone related to emergency care due to the hospital’s requirements, Tumbi, in general, focused on maternal and child health. So, for us, the goals of maternal and child health were achieved. However, other hospitals sent participants from different fields, such as nuclear medicine, which wasn’t directly related to maternal and child health. Regarding transparency, our institution would send the names of four individuals, and the selection process was based on applications, CVs, and relevant documents. They would call and interview the selected candidates, asking about their expectations and what they plan to do for their institution upon return. The selection was then made based on their responses. So, I think there was transparency because the institution would send names, and the project organizers would conduct the selection process.

**I: Alright. How would you assess the effectiveness of the way trainings were organized and supervised? Do you think it was satisfactory or you real wanted some things to be improved?**

P: In Korea?

**I: Yes.**

P: To be honest, for us in the beginning, there were a few challenges, but with time, they diminished. Any country you go to will have some challenges. They have a robust insurance system; all their patients are insured, making it difficult for a foreigner to interact with their patients for just a month. They need to be sure about your work and your insurance because if you accidentally harm a patient, they want to ensure they can address it. So, in the beginning, we mostly observed and practiced. For instance, as a radiologist, I could read images on the computer, but I wasn’t allowed to provide a report. Unlike those who went in subsequent years, they could read on the computer, and a supervisor would come, review, confirm, and agree on the report. In the end, it would be signed by both the supervisor and the participant. For us initially, that process wasn’t in place. We just had the opportunity to study the images on the computer without issuing reports. The supervisor would come and ask about what we observed, and they would provide additional explanations if necessary. So, it was more about self-study. In terms of patients, we weren’t allowed to touch them due to the lack of insurance, although they suggested that we could. However, because of that challenge, we raised concerns that in some areas, you couldn’t practice by observing alone; you needed to work. They addressed those concerns, and those who went later had the opportunity to examine patients and even practice image reading, administer medications, perform operations – all because it was a different program throughout the year, and they were covered by insurance. So, for us in the first three months, it was mostly observation.

**I: Here you mentioned about the expectations you had before attending the training/ fellowship programme in Korea such that even in the the interview that gave you opportunity to become part of the project, you were asked if there expectations wanted to meet at the end of the day. What was your feeling then after attending the training, did you feel like, your expectations were met or things turned to be different from what you expected?**

P: My expectations were the same, although our program would have been more beneficial if it were six months long. Those in the six-month program had the opportunity to practice, while for us, as I mentioned before, it was mostly about observation. We would observe how ultrasound is done, but we couldn’t operate the machines on patients since we were not allowed. So, in a way, I found what I was expecting, although not 100%. If our program had been longer, like the one-year or six-month programs, I believe we could have achieved even more—perhaps more than 80%, but at least 50/60% of the expectations were achieved.

**I: Suppose I am coming from one of the regions present in Tanzania, and for the first time someone brought me to Dar es Salaam. Imagine the feeling…I mean what it feels being exposed to Dar es Salaam with amazing amenities, lights etc. Then, I go back to my region or village, and someone asks me ‘what meant having got an opportunity to go to Dar es Salaam’, perhaps I will have a lot to explain based on how it feels and the like. Let’s talk about this in the context of our project. What it meant for you being part of the project as one of the participants? How would you have felt if you didn’t get this opportunity?**

P: Well, first, it’s an opportunity to go abroad and see what others are doing. I’ve studied abroad, but it’s different when it comes to working. I used to study abroad, but now I’m back working, and I got the chance to go there and see how they work and learn something new. When you go there and witness how they work, you wonder what Tanzanians might be lacking since they are humans just like us. They have two eyes and heads, so why can’t we do something similar? What I learned the most from that project, apart from the technical aspects, is their attitude and work ethic. You find that when you go to the office, others have already arrived by 12 noon, having reported at 12. They are time-conscious, and when it’s time to eat, everyone closes their computers to go and eat. They don’t waste time. In our setting, someone might take three hours to eat, but there, they finish quickly and return to work. Since they have an open office system, everyone is at their computer, and you won’t find someone playing cards on the computer in radiology. If it’s radiology, you’ll find an image on the screen, references, and someone preparing a report. So, that work ethic they have, even when I returned and went back to Tumbi, although we lacked equipment, I felt like I was in Korea. You feel like you’re at work, and you can’t just sit around chatting or listening to the phone. You work continuously. So, that work ethic, the spirit of work that they have, has had a significant impact on me, even in daily life. It’s not about moving slowly; you’re called to the office, and you go quickly. So, my visit to Korea had a great impact on me, not just in the project context but also in instilling that work spirit, working hard as if you’re working for yourself, not just because you’re employed and receiving a salary.

**I: You may have talked about this in our conversation, but maybe you have something to add up. When you started, you mentioned that the project aimed to build the capacity of those who participated. Looking at it, do you think the project achieved what it intended?**

P: I believe it achieved its goals to some extent, although not 100%. When you look at healthcare workers, we are many, but those who had the chance to go were not as many. So, considering the goals and the ongoing process of disseminating the knowledge gained by going to different regions, it’s one of the ways we’ve achieved our objectives. For example, we now write papers based on what we learned in Korea, and we look at what we want to do in the regions. In the past, we went to Lindi, gathering people from all districts of the Lindi region to teach them how to handle equipment. We managed to revive many oxygen concentrators that were thought to be useless, but after servicing and training on maintenance, they were revived, and people took them back. So, the goals of the project are being achieved, especially through this effort to send those who went to train others in different regions. It helps in disseminating the knowledge we gained.

**I: Are these seminars still ongoing?**

P: Yes, they are. For instance, our group recently went to Shinyanga, although they went to focus on emergency care. They go every year, each time with a different theme depending on the group.

**I: I would also like to know how the project benefited you individually and the institution you were working for.**

P: Firstly, in terms of knowledge, there were many things we did in ultrasound that were half-done, either due to lack of equipment or insufficient understanding. When I returned, I could do more detailed work. For example, my obstetric reports became detailed, to the point that even doctors enjoyed them because I provided unexpected information. Sometimes, what you used to see as normal turned out to be pathology, and you suggested further tests. So, when I returned to Tumbi, due to space constraints, I couldn’t practice much. However, when I moved to Mloganzila, equipped with similar facilities to those in Korea, I started applying what I had learned. I could work with various imaging tools, not just ultrasound. We had mammography for breast imaging, and we could perform biopsies. So, the institution benefited significantly from the increased capabilities in ultrasound, and in breast imaging, we could provide services that were lacking before.

**I: Looking at it, do you believe the skills you acquired will continue to be valuable in the long run?**

P: Of course, when you learn something and practice it, the knowledge stays with you. If you’re not selfish and continue to teach your juniors, even if they didn’t go to Korea, they receive that education. So, you can keep building skills. As I mentioned, I received education, trained others, and continue to train others every year. Even though I’m retired, there’s still an opportunity because every year, we participate in activities to train people in different regions. So, the skills continue, and what we learned becomes a continuous process of teaching others.

**I: Now I want us to look at the project along with the aspect of sustainability. What are your thoughts on the continuity of the project activities and outcomes especially when KOFIH funding gets to an end?**

P: To be honest, without hiding anything, we face the challenge that some projects depend on the interest or movement of higher-ups. If those at the top get involved effectively and see its importance, the project may continue systematically. If not, even if the project ends, it might only last for a short time, and then it fades away. We who gained the knowledge will continue to share it, but those outside won’t benefit because these projects require funding. If those who initiated the project withdraw their support, it may fade slowly until it dies. So, any project needs funds. Our colleagues have made efforts, and as long as the government and relevant authorities see the importance of those who went to study in Korea, the annual gatherings to facilitate further training and knowledge transfer may continue, and the impact will be positive. However, if they decide to stop bringing people together and injecting value by facilitating trips for continuous education, then the project may decline.

**I: Anything else you would like to add or something you wish could be improved in this project?**

P: Well, regarding this project…

**I: Yes**

P: I think, besides sending individuals to Korea, it would be beneficial to conduct some of these activities here in our hospitals. They could bring experts. Instead of sending one person to Korea for six months or a year, you can bring an expert from Korea to a place like Mloganzila Hospital. They could teach many people simultaneously. You could train more people at once, such as pediatricians, radiologists, and other health professionals. That way, you could reach many people in a shorter time. So, if there’s an opportunity to improve, it would be to bring motivation by sending individuals to Korea but also bring experts here to teach and benefit more people.

**I: Alright. If I give you an opportunity to say as a conclusive remark, what would say especially on the things you liked most in the project and those you didn’t?**

P: (laughs) I don’t know what to say. I don’t think there’s anything I didn’t like. I liked many things, and even if there were things I didn’t like, I carried them for the benefit of the country. For example, when you send someone there, they have an age limit; they prefer individuals below 45. A person around 48 might think, “Why do they want someone below 45?” However, considering the laws of nature, we live knowing that whoever comes first is the first to leave. So, it’s better to send young people for more extended service before retirement. I went in 2010, and after 13 years, I’m retired. Meanwhile, young ones who went in 2012 will retire at around 20 years of service. That’s much better.

**I: Oh, my dear mother I thank you very much. I appreciate a lot for your time and cooperation.**

P: You’re very welcome.

**KII WITH CLINICAL EXPERT-TUMBI, PWANI**

| **Date** | 24/11/2023 |
| --- | --- |
| **Region** | PWANI |
| **Location** | TUMBI |
| **Language used** | Swahili |
| **Translated** | English |
| **Gender** | FEMALE |
| **Position** | Clinical expert |

**First of all, I would like to know from you how the activities and goals of the projects sought to address the needs and priorities of health care system and healthcare providers?**

P: Yes, indeed, this project aims to address health challenges, especially in the maternal and child health department where there have been many deaths. With the ongoing support and training provided by this project, particularly in short courses related to maternal and child health, there has been significant improvement. In the past, we used to experience a high number of maternal and child deaths, but since the inception of this project with its training and equipment support, there has been a significant reduction in those deaths. The knowledge we gained from the training has been beneficial. For example, for pregnant women, they supported us with machines called Electronic Fetal Heart Rate Monitoring (EFM) to monitor the condition of the fetus in the womb. This has been instrumental in quickly identifying distress situations and providing prompt assistance to the mother. Previously, we used to rely on traditional methods of listening to the heartbeat, but these new machines have greatly supported us. Additionally, the training has been crucial because a significant percentage of mothers experience bleeding during pregnancy. The project has assisted us in dealing with such cases effectively. Personally, I may not have encountered many cases like that in my area, but the methods I learned during the training are immensely helpful when presenting cases here.

**I: You've explained in another way how the project has tried to address the priorities of healthcare providers in your department as a doctor. Do you also believe that the project aligns with the priorities of the country?**

P: Yes, it aligns with the country's priorities because the overarching policy of the country is to reduce maternal and child deaths. We can see an improvement in national statistics when comparing past years to the present. So, this project has made significant changes.

**I: Looking at the KOFIH project, which you've had the opportunity to attend and participate in training, how do you compare it to other healthcare projects in the country? Do you see any areas of convergence with other projects implemented in your area and the country at large?**

P: I'm not very familiar with other projects and how they operate, but when it comes to maternal and child health, it's a sensitive issue. When you address the mother, you touch the entire community, and the child is also part of that community. So, when a mother leaves, there is a significant gap. From what I see, this project is specifically focused on this area, and it touches the community at large.

**I: Do you see any differences between this project and other projects notwithstanding the fact that all aimed to improve health, especially in Pwani Region?**

P: In this project, despite providing training, they also support by supplying equipment and tools. That's something significant I have noticed. They not only train us but also provide us with the necessary tools, so you can't say you've been trained but lack the tools. They make an effort to supply both training and tools, making our work easier, and we see positive changes.

**I: We have different health committees starting from regional, district and even at the facility levels. We have RHMT at the level of region, CHMT at the district level and other committees down there at the level of facilities. I wanted to understand how this project has considered the priorities of these committees. Do you think the project has tried to reflect the priorities of these committees?**

P: Yes, the preparation was good because people were involved. When you are involved, names are suggested. For example, during my period, there was an age limit, and if someone had never gone before, they were given the opportunity. So, they involved us, and names were suggested. After that, the process began. There were interviews conducted last year, done online. We went in person and sat for interviews with experts from Korea. They asked questions, and afterward, we received an email informing us that we got the opportunity. Other processes followed thereafter.

**I: Did you apply for the opportunity or how did it go?.**

P: We went, and it depended on the departments they wanted to sponsor, the areas they wanted to support. For example, at Tumbi, there were those from the orthopedic department and those from the maternal and child health department. In my year, I was from the maternal and child health department, and there was another person from orthopedics. We went for the interview, and they selected based on the specific needs of each department.

**I: Were you satisfied with how the participants were selected in terms of transparency**?

P: Yes, there was transparency. There was no secrecy; it was an open process.

**I: Which year did you go?**

P: I went in 2019.

**I: As a clinical expert, right?**

P: Yes.

**I: Okay, great. How about the issue of gender? Was it considered during selection?**

P: I think it did focus on gender because, for example, in our group, there were two of us – one female (myself) and one male. In the orthopedic department, there was only one participant, and he was male. So, I believe they try to balance the gender representation.

**I: Do you see the project as having achieved its targeted goals?**

P: In my opinion, yes, to a large extent. The goals have been achieved because the challenges I used to encounter have, for the most part, been addressed with effective solutions.

**I: Can you give an example?**

P: For example, as I mentioned earlier, we used to face difficulties in monitoring the fetal heart rate of a mother during labor. The machines they provided have made it much easier. When you observe changes, you know the baby's condition and can take immediate action to help the mother deliver safely. Also, in terms of ultrasound, we didn't have ultrasound equipment before, and patients had to be taken elsewhere. The project provided us with ultrasound machines, making it easier for mothers to know the condition of their babies without leaving the ward. This has significantly improved our performance and achieved the project's goals. Mothers now leave the hospital with their babies in good health.

**I: Despite the project having its goals, were there any personal expectations you had as a participant before you attended the training? And how such expectations were met after the training?**

P: I'm grateful that I achieved most of the goals I had set for myself, although not entirely. Cases of eclampsia, for instance, were not as prevalent there as they are here, but I still managed to learn about them. I observed their operations, and my mentor, the professor, was very supportive. I learned a lot from him, even during clinics where he guided me. In terms of ultrasound, I was delighted because I got to accompany them on ultrasound cases, something I hadn't seen much elsewhere. I learned a lot, especially in maternal and child health, gaining confidence.

**I: What did you find unsatisfactory or wish could have been done differently in the project?**

P: Well, in that setting, cases of bleeding during pregnancy were not as common, and practically I didn't encounter them. The professor explained them theoretically, but I didn't have the opportunity to see and learn practically.

**I: How would you describe the significance of being part of this project as a participant who attended the training sessions?**

P: I am happy and proud to be part of this project because I have progressed significantly compared to when I first went.

**I: And perhaps, if you hadn't had the opportunity to attend these training sessions, how do you think it would have been?**

P: I would have missed a alot.

**I: Alright. Maybe, doctor, as I mentioned earlier, there might be other projects being implemented different from this KOFIH project. I wanted to know, how activities of this project affected the implementation of other projects in your area. Can be negative or positive, please.**

P: I don't know much about other projects.

**I: Ok.**

P: But for this KOFIH project, I have seen it make significant strides because even as we attend those sessions and presentations for feedback, you can see how it has touched many parts of Tanzania. Many regions in Tanzania.

**I: I wanted to know if you are having let say, seminar sessions, workshops, meetings to share things related to this project including what you have learnt from the trainings.**

**P: Yes.**

**I: How does it happen? How do you conduct it?**

P: So, all of us who attended are sent emails. We meet, share feedback, and discuss what we've learned, such as the workshops we did in different places.

**I: Alright. How would you explain the efficiency of the training sessions?. It might be the accountability of the trainers, and those who were responsible for coordination of the sessions.**

P: Ah, here or?

**I: Ooooh, no, those people who have been managing the entire project at KOFIH.**

P: Oooh

**I: Were you satisfied with their efficiency in terms of managing the sessions and project activities?**

P: Well, their management is good. I say it's good because from the beginning, they start identifying, for example, things in the lab. They might tell you in your lab, there are certain things, like machines, or in a department, there are certain things. So, from there, I think they select gaps, those are the gaps they start helping with. Like when we were there, they asked if there's something in your hospital that, if we help you with, you can perform better? So, from there, they help you by supplying. So, at least, you can see that these people look at those gaps and fill them to help you.

**I: Let us now talk a little bit about the way things were handled. Was everything done on time? You can also talk about the allowance if it was provided on time or there were some sort of delays and complaints?**

P: They used to come on time at the beginning, I think. But I don't remember much. It's not like you go there and live without knowing what's going on; no, they are organized people. If there's something they think might be an obstacle, they say it early, maybe this will hinder the success of this exercise, but after some time, it will succeed. They handle it that way.

**I: You might have mentioned this, but maybe if you have something to add. Can you tell me how this project has benefited you individually as a healthcare provider. Also, talk about the benefits for the institution you are working for.**

P: For me, personally, it has helped me to have confidence in my work. I can handle a case like this, know what to do practically. When it comes to a point where I don't have something to do at the moment, I ask for help or forward it. And for the institution, it has helped because if a problem arises with a mother or child, the whole institution appears. If there is a perception that the performance is not good or it tarnishes the institution's reputation. So, it also gives the institution respect. People say, "Ah, if I go to deliver at that center, I come out safe," you know.

**I: Speaking it from the past experience you had, do you see any differences between what you have now and before attending the training?**

P: Yes, indeed. In the beginning, we faced a lot of challenges, and it was really a challenge. When you sit there, you are anxious about what to do. But now, at least, we have a monitor, and there's no problem. Even the mother herself, you can explain to her, "See, your child's heartbeat is normal, and your child is fine. Don't worry." It helps the mother to stay calm, and there's no stress.

**I: What would you consider as the unexpected results of this project?**

P: What do you mean?

**I: I mean, a project may have achieved beyond expectations and still being ableto have achieved far beyond what is expected. Do you see any impacts of the project that were unforeseen by the project? .Perhaps, the project did not expect to gain this particular benefit, but it happened. So, I am asking if there are any unexpected results.**

P: Ahaaaa. Maybe I should say an increase in clients. Because now, they see that the services are going well, they are treated well, and they increase. You know, mothers tell each other, "Husband, go there, they welcome you well, they have good services, and there are tests, etc."

**I: Doctor, normally we would want to see the project activities and outcomes continuing being sustainable, meaning, they do not come to an end even when funding does so. In your view, looking at the activities and outcomes of this project, do you see any indications of being sustainable?**

P: Well, in my view, I see them as sustainable because the equipments are there, and it's checked, inspected, and maintained. Even if the project dies, they will still be there.

**I: Apart from the equipment, what else do you consider to be an indicator of the sustainability of this project?**

P: Job performance. Now, you've gained that skill, you keep developing it; it doesn't expire.

**I: Knowledge doesn't expire, exactly. What do you think should be prioritized sto improve and enhance the sustainability of project activities and outcomes?**

P: Well, for me, what could be improved is maybe if I had two pieces of equipment, I could continue by adding at least one each year. This is because the number of our clients who visit us is increasing. If the government could allocate a budget periodically for the installation of certain machines, even if the project phases out, at least after a few years, they would be there in abundance.

**I: Speaking of the skills and knowledge you've acquired through training, do you see yourself being able to utilize them in a long run? DO you see yourself continuing to use those skills for a long period of time?**

P: Absolutely, absolutely.

**I:As we are winding up doctor, I was curious to know if government institutions or other stakeholders will be able to sustain the project activities and outcomes?**

P: I think it's possible. Our government's procedure is to allocate a budget, buy the equipment, and maintain it. I see many hospitals having this Biomedical Engineering (BME) department, where people trained in equipment maintenance work. Especially in regional and referral hospitals, I see that now. So, I believe these things will also continue to exist because the equipment needs maintenance.

**I: Alright. Do you have anything else you would like to share with us that you feel we didn't discuss here but is related to our topic? Any suggestions, opinions, anything?**

P: Well, I just want to thank this project. It has helped me a lot. The beauty of it is that in these clinical expertise skills, knowledge doesn't wear out. It continues to grow year after year, and others learn from you. Instead of being educated alone, you find others also continue because they have learned from you, who has already learned. So, it becomes something continuous.

**I: Alright. I want to thank you very much doctor, for your cooperation and time. Thank you very, very much**.

P: You're welcome, and thank you too.

**KII WITH A NURSE-TUMBI, PWANI**

| **Date** | 24/11/2023 |
| --- | --- |
| **Region** | PWANI |
| **Location** | TUMBI |
| **Language used** | Swahili |
| **Translated** | English |
| **Gender** | FEMALE |

**I would like to know from you how the activities and goals of this project are aimed at addressing the needs and priorities of healthcare providers.**

P: Indeed, regarding medical equipment, I can say the goals have largely been achieved because understanding medical equipment and maintenance has personally helped me even identify issues in various regions in Tanzania. I was sent by the ministry to Dodoma in the Mpwapwa district, and in 2020, I went to Mtwara for equipment repairs. So, it has helped me understand how to repair medical equipment and maintain them, aiding in providing quick solutions, especially during nighttime emergencies.

**I: Yes, and in your healthcare profession, you may have priorities and needs, among other things enhance your performance. How has the project reflected them?**

P: This project has targeted and addressed those priorities I had because, with faulty medical equipment, proper patient care is hindered. If medical equipment is defective, the treatment of the patient is incomplete. Understanding the equipment helps the healthcare provider address the patient’s issues promptly, and that is the primary goal – to aid patients quickly.

**I: Right. So, in your field of biomedical engineering, you attended training in 2010, 2011, and 2014, three times. Right?**

P: Yes, Biomedical Engineering.

**I: In the field biomedical engineering, what aspects did the training focus on?**

P: It covered medical equipment, for example, basic monitors and how they operate. Also, we learned about the manufacturing process, even in industries, like how X-rays are produced in factories. The basics are crucial; when a breakdown occurs, you can quickly troubleshoot. Especially in situations where it’s challenging for a Korean to come here for repairs, it’s better to have local experts trained to handle such issues.

**I: I’d like to know how the project managed to meet the needs and priorities of healthcare providers.**

P: The initiator, Dr. Lee Jong, provided healthcare services in hospitals, so the project targeted the people involved in hospitals. For instance, biomedical engineers were targeted because they are directly involved in the medical equipment process. I’m not sure about the exact process, but during my time, interviews were conducted online, and face-to-face, and individuals were selected to go for training.

**I: How many people were selected after the intervew?**

P: They wanted names of all people in the relevant department, and then they started the interview process. The number depended on the demand each year.

**I: Do you think the project’s activities and outcomes align with the country’s priorities? Each country has its priorities, and for example, in the health sector, there might be specific priorities. Did the project align with the country’s priorities?**

P: When we talk about the country’s priorities, every government has its own, like education and health, but health is crucial because, without good health, nothing else can progress. So, I believe it aligns with the country’s priorities because if the citizens are healthy, everything else falls into place. Health is the foundation; without it, nothing can be achieved.

**I: How does this project differ from other health projects in the country? What makes it unique or distinct?**

P: One thing I can mention is that health is vital, and this project, particularly focusing on medical engineering and equipment, is crucial. When a patient goes to a hospital and is told they can’t be treated due to faulty equipment, it’s a serious matter. Having experts who can repair these devices locally is important, and that’s what makes this project unique.

**I: What gives it that uniqueness that you see, which sets it apart from others?**

P: One thing is the emphasis on medical engineering, particularly on medical equipment. When a patient faces challenges, and they are told treatment is impossible due to faulty equipment, it’s a serious issue. The ability to have technicians who can fix these devices locally is vital.

**I: Do you think the project aligns with different committees speaking of those present at the regional, district and at the levels of the health facilities?**

P: Yes, there is a connection. For example, at Tumbi Hospital in the Pwani region, the Koreans support medical equipment in health centers. In Mlandizi, initially in 2010, they started supporting Tumbi Hospital with Korean medical equipment. They started with the coding for biomedical engineering, and they expanded to other health centers. Now, I believe they cover a quarter of the country. Biomedical engineers trained in Korea come from various regions like Mwanza, and this growth is due to the availability of medical equipment.

**I: Looking at other projects being implemented in the country or your region in specific, what is feeling regarding this project being able to align with the priorities and activities of other projects? Is there any point of convergence or divergence?**

P: Yes, I can say it aligns. To me, these individuals came as sort of saviors for medical equipment in Tanzania. There is a significant contribution, around a hundred percent, because in health centers like Tumbi Hospital or Mlandizi in Pwani, there’s a part where Korean medical equipment supports the Tanzanian government’s equipment. It’s something unique; it’s like a fusion between Korean technology and government-owned equipment, so it plays a crucial role in equipment availability.

**I: Do you think the project intended to address the needs and priorities of healthcare workers?**

P: It has focused in such a way that in terms of medical equipment, as I mentioned, one’s capital is good health. Therefore, it has focused to the extent that the equipment reaches on time because there was a period before this project when there were some devices, but they were few. The Biomedical Engineering course, as I mentioned, did not exist in 2010, but now, after five or six years since the project started, the challenges and the manufacturing of medical equipment from technicians going to Korea have been very helpful. That’s why the university was established in Dar es Salaam and Arusha. If I’m not mistaken, that’s where this course is offered, and these training programs have been initiated. So, I can say they are the founders of the challenges of medical equipment, which now serve as a refuge to help patients.

**I: Alright, in general, what are your opinions on the training the project. Looking at the entire process of getting the participants, I mean selection process?**

P: I can say the preparation is not bad nor very good. I can say it’s in the middle because during that time when we were preparing and selecting how these technicians would be chosen, they used to visit every hospital, starting from Tumbi, to see the challenges and needs at Tumbi, and the Department of Medical Equipment. The technicians came with the intention that their number would match the number needed, and they took them to the relevant training. So, I can say it has met the requirements. It’s not bad, but it’s not excellent because now I hear there’s an interview process for people to attend biomedical engineering courses. Currently, there’s an interview, and if you pass that interview, then you go for the course. In those days, there used to be a visit to every hospital to provide assistance and take a representative from the hospital for the training and bring them back. So, that person becomes a significant help when assistance comes from them. If there is any breakdown, communication is established, and if a breakdown occurs, they send someone to fix it.

**I: So, you were interviewed. Is that what you are trying to say?**

P: We had an interview, but the interview was limited to the technicians at that specific place. For example, if there are six technicians, they conduct interviews among those six. But nowadays, they conduct interviews with people from different places, and those who are selected come from the areas where they themselves have identified.

**I: How would assess the content of the training?**

P: Honestly, it’s good, and it has helped a lot. For example, we were sent by the ministry to different regions to conduct inventory, inventory of medical equipment, and short-term maintenance of machines. Personally, I got to know Dodoma through the Korean training. I got to know Lindi and Mtwara because of these Korean training programs. Also, getting to know Korea itself because due to certain equipment, I got to know Korea in 2010, 2011, and 2014 because of medical equipment. So, there is a great need or great thanks to this project for the way it helps us to know these places I have been through because of their contribution.

**I: Looking at the aspect of gender, do you think this project has promoted gender equality. Through its project activities, do you think both male and female participants are given equal priority in terms of resources and training opportunities?**

P: Gender equality exists; there is no bias. They favor neither gender. There is normal equality.

**I: How many participants were selected for your intake/ cohort? if you can remember**?

P: In 2014, we were six Tanzanians in the biomedical field.

**I: How many were women?**

P: There was one woman.

**I: And what are your thoughts on the project continuing to sustain its activities and outcomes even when the funding ends?**

P: I think it does, but there is still room to focus more on rural areas. I believe focusing more on rural areas will be very helpful. Currently, the government’s plan is for every district to have quality medical equipment. The burden on regional and district hospitals is reduced because medical equipment is at the district level and health centers are strong with quality equipment. So, focusing more on rural areas will reduce the burden on regional and district hospitals.

**I: Were you satisfied with the level of transparency in the selection process and information sharing ?**

P: Personally, in biomedical engineering, as I mentioned, after the interview is conducted, we were told that there are six technicians, and there will be an interview to go to Korea. So, it would be clear to everyone, and we received calls where you see an unknown number, and you check, and you are told, “I am so-and-so from Korea, and I came there for medical equipment.” The interview starts, and those selected go for training. So, it was clear. I don’t know about now because since 2014, it’s been a long time.

**I: When you were selected to be part of this project as a beneficiary, did you have any expectations? Do you think your expectations were met? What kind of training did you expect to receive, and did you receive it as expected? What do you think was good and not?**

P: I can say, personally, my expectations were met to a large extent. Because the issue of medical equipment has been something I liked since I was young. That’s why I started with an electrical engineering course. After completing the electrical engineering course, I started working on medical equipment because I was involved during that time. Personally, I can say it helped me a lot because even the equipment, for example, the hypas ion monitor, how it works, the entire sequence of how it works, how to assist a patient, it has helped me understand this device more easily.

**I: What does it mean for you to be part of this project? What does it mean for you to be one of the alumni?**

P: Personally, I interpret it as some form of salvation because I was there to help medical equipment at that time. For example, if a device breaks down for some time, I can quickly repair it and assist a patient. So, for me, the meaning is that I was a savior in a way because I was there to help with medical equipment. If he wasn’t there in that particular place , or if he didn’t know, a doctor could treat a patient, and the device could give false results. But there would be no technician available. So, it’s essential for a technician to be there. Also, in understanding these devices.

**I: How would you feel if you were not part of this project?**

P: Honestly, I would feel hurt. I would hurt because it’s something I liked so much—to repair medical equipment. So, if I wasn’t selected, I think they would have chosen others. It would have given me a hard time, and it would have been difficult for people to understand through me. Instead of me learning from Korea, it would have been challenging.

**I: Okay, do you think the activities and outcomes of this project affect the implementation of other projects in your area?**

P: I don’t think it affects because health issues are a priority, and equipment is a priority. So, each one is implemented within its timeframe. So, I don’t think it affects.

**I: How effective do you think those who are responsible for the implementation of this project were? How well did they manage or operate the project’s activities?**

P: There is effectiveness in terms of how you see medical equipment coming and being maintained on time. It has been efficient enough. If the equipment was coming just like that to the hospital—for example, the equipment would come, and if it breaks down, the technicians are indeed there, and they repair it. So, it seems effective enough because the equipment comes on time, is used, and if it breaks down, it’s fixed by the technicians. They are significant contributors in addition to conducting this project. They send people for training and, at the same time, respond promptly by sending spare parts when there’s a breakdown.

**I: Were the project activities done in timely manner. For example, you can talk about allowance provided. Was it provided on time?**

P: It happened on time because as soon as you arrive, you are given money for sustenance, and halfway through after two weeks, you are given money for sustenance, and when you are leaving, you are given money for sustenance on time.

**I: Now, let’s look at the impact of the project. What overall impacts do you think the project has achieved?**

P: Yes.

**I: How so?**

P: In terms of medical equipment, the equipment they bring, for example, to Tumbi Hospital and Malandizi. When the equipment malfunctions, you contact them, and they promptly send spare parts. So, repairs are done, and the equipment remains original, continuing to be used as usual. Therefore, there have been benefits.

**I: Have there been any barriers that may have affected the intended results of the project?**

P: Honestly, I haven’t seen any obstacles.

**I: How are you benefited by this project as an individual person and healthcare provider?**

P: I have benefited in two ways. First, learning about Korea – without this project, I don’t think I would have known about Korea. Secondly, I learned about various regions. For example, I had been sent by the ministry to take inventory of equipment in places like Dodoma and Lindi. Another thing I’m proud of is receiving an email from Korea recently, asking me to take inventory in Mlandizi and other regions. A month later, the Koreans visited Tungi Hospital, and Mr. Park came. We had communicated with him before, asking him to be present at Tungi Hospital. So, he came, and we conducted the inventory. I take pride in doing the inventory because they taught us how to do it, the basic maintenance, and I have benefited from that.

**I: How has this specialized knowledge of medical equipment benefited you?**

P: I can say that, like any other employee, when called to learn about medical equipment, it’s a specialization. It’s truly a specialization in medical equipment, but I feel we are on the right track.

**I: Do you think they have also benefited the institution?**

P: I can say it has benefited significantly because previously, before this training, the equipment would malfunction, and it would be stored until a technician from another place came to repair it. Therefore, it saves costs. As an employee, if the equipment breaks down, and you have gone through this training, you repair it, and it continues to be used for patients. It saves time between repairs and the waiting time for a technician to come.

**I: Are there any unexpected results from the project? I mean those which were not anticipated.**

P: I can say yes or no because, in expectations, when you are producing medical equipment on time or producing high-quality equipment, patients tend to flock to that hospital. A place with poor services won’t attract many patients. So, if high-quality equipment from Korea or elsewhere, with specialists in that equipment, comes in, it attracts patients. They come knowing there are quality facilities, and the equipment is being used promptly.

**I: Do you think project activities and outcomes will continue eve when the funding from KOFIH ends?**

P: The results will remain positive because the knowledge they have given us must be used. Even if the project stops, the fund is still there, and they will use that fund to continue supporting equipment and professionals. So, even if they stop, the fund they have set up is there, and it is their goal to support equipment and professionals. If they stop, even if it falters, the knowledge is there, just like when a person ages, the knowledge doesn’t vanish.

**I: What should be prioritized to ensure sustainability of project activities and outcomes?**

P: Prioritize alumni groups by increasing the budget. Increase the budget so that more people can be reached quickly. The existing budget should not limit them. At least, reach more people at once and quickly. If possible, twice a year, not just once, because our country is vast, and there are many regions. They should work twice a year to help people, not just once a year.

**I: Will you continue to use the skills and knowledge gained from this project even when the project comes to an end?**

P: Yes, as I said, knowledge doesn’t fade.

**I: Looking at governmental institutions and other partners who may have been involved in the project’s implementation, do you think they will continue to run project activities and outcomes when the project ends?**

P: I doubt it because some hospitals I have visited, I’ve seen KOFIH equipment and government equipment. I’ve already seen this situation, so when you inquire and learn why the equipment is in this state, they will continue. But as I reiterated, these alumni help a lot because whenever they visit a specific region, they find out why certain equipment is in a particular condition, and with the funds they have, they can fix that equipment on time.

**I: Is there anything else you would like to add or discuss that perhaps we haven’t touched upon, and you’d like to mention?**

P: I can say that KOFIH has enlightened me because it has made me first know about Korea and some regions. The skills and knowledge it has given me are beneficial. I know about Korea and various regions, and now, I can be called from anywhere, even from Mwanza, for assistance. I have gained this trust, and they have placed me like a mark that doesn’t fade quickly.

**I: Well, thank you very much for your time. I appreciate your cooperation and the insights you’ve shared.**

P: You’re welcome. Thank you.

**KII WITH CLINICAL EXPERT, TUMBI, DAR ES SALAAM**

| **Date** | 24/11/2023 |
| --- | --- |
| **Region** | PWANI |
| **Location** | TUMBI |
| **Language used** | Swahili |
| **Translated** | English |
| **Gender** | FEMALE |

**I:** **First of all, how has this project aimed to address the priorities or challenges of healthcare workers?**

P: Yes, indeed, this project aims to address the challenges of maternal and child health. There were many maternal and child deaths, and this project continues to support by providing short-term training related to maternal and child health. It has been of great help. Previously, we experienced many maternal and child deaths, but with the training and equipment provided by the project, there has been a significant reduction in such deaths. The project has provided tools such as UCCTG machines to monitor the fetal condition, which has been very helpful in detecting emergencies and providing timely assistance.

**I: You’ve described how the project focused on the priorities of healthcare workers. Do you also think it aligns with or addresses the country’s priorities?**

P: Yes, it aligns with the country’s priorities because the main goal is to reduce maternal and child deaths. When you look at the national statistics, there have been significant changes since the project started. Therefore, it aligns with the country’s priorities and brings positive changes.

**I: Comparing the KOFIH project with other projects in the country, do you see similarities or differences**?

P: I’m not sure about other projects, but in terms of maternal and child health, this project stands out. It directly addresses critical issues related to maternal and child deaths, focusing on a significant aspect that affects the community.

**I: What do you see as the main difference or unique aspect of this project compared to others?**

P: The significant thing is that, besides providing training, this project also supports by providing tools and equipment needed. It’s not just about training; they ensure that we have the tools to effectively implement what we have learned. This makes a significant difference because, after training, we also have the resources to apply our knowledge effectively.

**I: We have committees at various levels, at regional level we have RHMT committees, at district level we have CHMT committees, and at health facilities we have health committees too. Did the project align with the priorities of these committees?**

P: These committees play a crucial role because they identify gaps and needs. For example, these committees propose solutions, and KOFIH helps implement these solutions. They collaborate to ensure identified needs are met. It’s a collective effort, and the committees have made significant contributions to planning and implementing strategies.

**I: Let’s look at the selection process, I mean how participants were obtained. How would you comment on the transparency during the time of selecting participants? Were the criteria clear, and were people satisfied with the participant selection process?**

P: Yes, there was transparency. The process involved recommendations and advice, followed by interviews. I went through that process and was informed by email when I was selected. So, there was no secrecy, and the participant selection process was transparent.

**I: How satisfied were you with the participant selection process, considering the aspect of transparency?**

P: I was satisfied because it was a transparent process, and there was no hidden agenda. It was clear, and communication was good. I had no issues with the participant selection process.

**I: Did the project consider gender equality in terms of participant involvement? How was the gender balance in terms of participation?**

P: Yes, I believe there was gender equality. For example, when I went, we were two – a female and a male representative. There was gender balance. In orthopedics, where there was no female representative, it was due to the candidates available at that time. So, I see a mix of genders, and it appears to be inclusive.

**I: Do you see the project achieving its goals as targeted?**

P: From my side, I believe the project has largely achieved its goals because the challenges I encountered seem to have substantial solutions now.

**I: Can you provide an example?**

P: For example, as I mentioned earlier, we had difficulties monitoring fetal heart rate during labor every few minutes. However, with the machines provided, it has become much easier. Now, when you see a baby in distress, you can take immediate action to assist the mother in delivering her baby safely. These improvements in equipment, such as ultrasound machines, have made a significant impact, helping us easily identify and address issues. It has simplified our work, and mothers are leaving with healthy babies.

**I: I suppose that you had your expectations before attending the training. Could you share if those expectations were met?**

P: I am grateful for what I learned and experienced during the training. While I didn’t achieve all the goals I set for myself, I did learn about conditions like Eclampsia, saw how they perform surgeries, and gained valuable insights. I was particularly pleased with the ultrasound training, seeing cases that I hadn’t encountered here. It enhanced my confidence.

**I: Is there anything specific that you were dissatisfied with or was different from your expectations during the training?**

P: In terms of cases, I wish there were more instances of conditions like maternal bleeding during pregnancy, which are not prevalent here. Practical exposure to such cases would have been valuable.

**I: If you had not attended this project, how do you think it would have affected you?**

P: I would have had some shortcomings because the exposure and experience gained were significant.

**I: Maybe there are other projects being implemented in this area apart from this one. Do you see activities and outcomes of this project affecting the implementation of other projects being implemented? Have you observed any positive or negative effects, or do they seem to run independently**?

P: I cannot speak much about other projects, but for the KOFIH project, I have seen significant impacts. During our feedback sessions, it is evident that the project has touched many areas of Tanzania and influenced various regions positively.

**I: You mentioned that you are sometimes having meeting sessions to discuss issues related to the project. How often does this happen and how do you go about?**

P: We communicate through email. All participants receive emails, and we share feedback on what we have learned or accomplished. We discuss workshops or visits to different places, fostering collaboration.

**I: How do you assess the effectiveness and accountability of those who manage and oversee the project? Were you satisfied with their management and accountability?**

P: The management was good. They initiated discussions early on, identifying gaps and needs. They were responsive, supplying resources according to identified needs. They had a systematic approach, and I believe they were effective in managing the project.

**I: In terms of program timing and financial disbursements, were these issues handled well, or were there delays and complaints?**

P: The programs were timely. Initially, there may have been some issues, but they explained the reasons behind any delays. They provided allowances upfront and had a systematic process. There were no issues in terms of timing and financial support.

**I: Based on your experience, how has the project benefited you personally and your institution in terms of your role as a healthcare provider?**

P: Personally, it has boosted my confidence in performing my duties. I am more certain in handling specific cases, knowing what steps to take. For the institution, it has helped maintain a positive reputation. When a mother says she is going to Tumbi Hospital to deliver, there is a sense of confidence that they will leave with a healthy baby.

**I: So, comparing your past experiences before attending the project, do you feel that you’ve gained something overall?**

P: Absolutely, in the beginning, we faced many challenges, and it was stressful. Now, at least, we can monitor things, reassure mothers, and use ultrasound to address concerns. It has reduced stress for both healthcare providers and mothers.

**I: Have there been any unexpected outcomes from the project that you didn’t anticipate but occurred after project activities were completed?**

P: Are you asking about…?

**I: You might have had certain expectations before attending the training. Do you think there are any additional benefits that came about unexpectedly from the project**?

P: Well, I’d say there’s been an increase in patients, especially because they see that services are going well. Mothers are increasing, and with reliable tests…

**I: When you reflect on the project activities and outcomes, do you see them as sustainable, continuing even after the end of the project?**

P: Yes, I believe it’s sustainable. Equipment is regularly inspected and provided. Even if the project ends, the services will continue.

**I: Besides the health facility equipment provided by the project , what else do you think will make this project sustainable?**

P: Work performance. Once you acquire skills, you can continue developing them. Knowledge doesn’t expire.

**I: So, in your opinion, what areas should be given more attention to improve the continuation of the project outcomes after it ends?**

P: For me, continuous investment, like getting two more of these… (26:2) every year, and gradually expanding based on the growing number of clients. The government could allocate a budget periodically to maintain a steady supply.

**I: Considering the skills you have acquired, do you see yourself using these skills for a long term/ in a long run even when the phase out of the project activities reaches?**

P: Absolutely.

**I: Thank you for sharing your insights. Are there any governmental institutions or other stakeholders who have been involved in the project, and do you think they will continue these project activities when KOFIH funding reaches to an end?**

P: I think it’s possible. Our government allocates budgets for equipment and maintenance. Many regional hospitals already have Biomedical Engineers, especially in referral hospitals. It helps in sustaining these facilities.

**I: Do you have anything else you’d like to share, maybe something we didn’t discuss related to the topic? Any suggestions, opinions, or anything else?**

P: I want to express my gratitude to this project. It has been very helpful. The great thing is that with clinical experts, knowledge doesn’t get lost; it continues for years. Others learn from you, and it becomes a continuous process.

I: Perfect. We appreciate your cooperation and time. Thank you very much.

# **KII WITH KOFIH BENEFICIARY**

| **Date** | 20/11/2023 |
| --- | --- |
| **Region** | Coastal |
| **Location** | Tumbi Referral Hospital |
| **Language used** | Swahili |
| **Translated** | English |
| **Gender** | Female |
| **Position** | ANO |

**I: As I introduced myself, I am here to conduct a short research or evaluation on the KOFIH project. As one of the beneficiaries, I would like to ask you a few questions.**

P: Welcome.

**I: Thank you. Let us go straight to the questions. These questions focus directly on the KOFIH fellowship and the training you received in Korea. Are you ready?**

P: Ready.

**I: Great. To begin, please introduce yourself, your name, and your position at Tumbi Hospital.**

P: Alright. My name is Lilah Godwin Kimambo, an assistant nursing officer working in the maternal and child health department, specifically in the parent surgery theatre. I’m also the assistant in charge.

**I: Thank you. Let us proceed. These questions aim to evaluate how well the project aligns with the needs and priorities of healthcare providers. Do you feel the project’s activities and goals were aligned with the needs and priorities of healthcare providers?**

P: Yes, the project aligned well with the needs of healthcare providers. It assisted us by providing knowledge about some equipment we had but didn’t know how to use. It also helped us understand the use of certain tools. The project supported us in the first-line management of equipment, making it possible for nurses to handle basic maintenance. Additionally, it improved our services, especially in maternal and child health.

**I: Excellent. Do you believe the project selected the right beneficiaries? Were those who went for training the right individuals?**

P: Yes, they chose the right beneficiaries. If you were going for maternal and child health, they selected healthcare workers from the corresponding departments.

**I: Was there anyone you think should have gone or someone who went but might not have been suitable for their position at the facility?**

P: They tried their best. There was a period when they seemed to pause the selection, but they later resumed. The individuals selected were generally beneficial when they returned.

**I: How much did the project ensure the various needs of healthcare providers were considered or given a space during the project’s preparation and implementation phases?**

P: The project did consider our needs, even enabling us to conduct in-house training.

**I: If you could elaborate on your needs and whether the project addressed them during its design and implementation?**

P: For those of us who went, we had to base our knowledge on the training we received, which was over ten years ago. There haven’t been updates or further training since then. It would be helpful if there were follow-ups or continuous training sessions.

**I: How well did the training reflect or align with the country’s priorities?**

P: It aligned well because we went for maternal and child health programs. The knowledge we gained directly applies to the priorities of our country, especially in maternal and child healthcare.

**I: Were there similarities between the KOFIH project and other existing projects in your area or the country in general?**

P: Yes, there were similarities, but KOFIH stands out because they provided both education and support, including equipment and infrastructure.

**I: How did the support from KOFIH complement other healthcare projects?**

P: KOFIH’s support was significant, especially in the construction of the maternal and child health building. They also supported us with equipment, including neonatal intensive care unit (NICU) facilities.

**I: Are there other partners contributing to addressing maternal and child health challenges in your area?**

P: No, there are no other partners involved in this specific area.

**I: Concerning the Council Health Management Teams (CHMT) and Regional Health Management Teams (RHMTs), to what extent has the KOFIH project reflected or aligned with their priorities or plans?**

P: CHMTs and RHMTs have been supportive, even enabling us to conduct in-house training. The project has complemented our efforts.

**I: Has the KOFIH project been successful in addressing critical needs of the healthcare workers? Has it been able to solve significant priorities of the healthcare providers?**

P: Yes, the project has successfully addressed critical needs. For example, we lacked a neonatal intensive care unit (NICU), and the project helped us set up one. It also facilitated the creation of a dedicated parent surgery theatre.

**I: Could you mention specific priorities that the project has been able to address?**

P: One major achievement is the establishment of a NICU, which was essential for providing intensive care to newborns. Additionally, the project enabled us to have a dedicated surgery theatre for parents.

**I: Let us switch to the second part about effectiveness. What are your opinions on the training provided by KOFIH? Were the preparations satisfactory, and how was the selection of the participants?**

P: The preparations were good. There was no specific requirement to fulfil during selection.

**I: Can you put it even in English?**

P: You mean, there was no, let us say, bias, meaning they would choose anyone, but from the relevant department? It’s not like we wanted a nurse or doctor from the maternal and child health department; they chose someone from the relevant department, someone they saw as knowledgeable, someone who could have an impact when they go—this is what they were doing. And the preparations for the journey and the entire program really gave us sufficient preparations from the time of travelling until arrival. Throughout the program, we didn’t encounter any problems.

**I: How about the training content?**

P: Well, the training content was good, but sometimes they would tell us to look for materials ourselves in their libraries when we were there. Somehow, it was a bit difficult for us, and…

**I: Why was it difficult?**

P: You would go to the library, and the attendants there at that time didn’t speak English well, so even if you needed help, you couldn’t find someone to assist you. Otherwise, other things were going smoothly.

**I: Okay, got it. What are your thoughts on the project’s efforts to achieve its goals, and the goals?**

P: We thought they should continue, not leave us. They should continue, maybe with another program, like taking our colleagues for further studies or if there’s a new initiative, they should update us. This is what we were expecting because things change; what you studied three years ago, now there are many updates. So, you’d be holding onto what you learned, but there might be new things.

**I: Now, suppose that the project has ended today, do you feel the project achievements or goals set by the project will be sustainable?**

P: They will be sustainable.

**I: Why?**

P: Due to education they provided us, and we are practicing it, we have trained others, so it will be sustainable.

**I: Okay. What is your feeling about the training’s impact?**

P: Mmm! even the training content was good. Sometimes they would ask us to find materials in their libraries, which was a bit challenging for us. Other than that, everything else went well.

**I: Why was it challenging?**

P: You would go to the library, and the attendants there at that time did not speak English well, so even if you needed help, you could not find someone to assist you. Otherwise, other things were going smoothly.

**I: Okay, understood. What were your expectations before attending the training, and to what extent do you think the project met your expectations? Let us start with your expectations. What were they?**

P: I expected that when I went there and returned, I would have improved in providing care for mothers and children. When I returned, I was able to meet those expectations by providing emergency care for mothers and children.

**I: What were you satisfied with during the program, and what dissatisfied you?**

P: What did not satisfy me was not getting a room to care for patients, in Tanzania, you could care for a patient and provide services. But there, it was more of watching; we couldn’t practice hands-on patient care.

**I: Okay. Anything else that dissatisfied you?**

P: No.

**I: What about what you were satisfied with? The positives of the project?**

P: The positives were during the theoretical part of the training. They provided good lectures; however, in terms of practical application, we couldn’t practice much.

**I: Okay. Overall, how do you feel about being part of the KOFIH project? In your opinion, what role do you see yourself playing in the project?**

P: I feel good; I am proud. I’m proud because of the knowledge I gained. With knowledge, you have a certain power. Knowing something allows you to do things, and you know where you acquired it. So, that’s why I feel proud.

**I: Why do you feel proud?**

P: Because of the knowledge I gained. You know, with knowledge, you have a certain power, and there’s something you can do when you’ve acquired it from a certain place. So, that’s it.

**I: Let us assume you did not get this opportunity to attend the training; how do you think your performance would be now?**

P: I think it would be at a lower level.

**I: Why?**

P: I would have missed out on many things, so my performance would not be as good as it is now.

**I: Okay. Let us move on to the efficiency part. Starting with the project activities, how do they match or affect the implementation of other similar projects funded by different stakeholders?**

P: They do not affect them.

**I: Why do you say that? Are there no activities that overlap or** **influence each other?**

P: And this project?

**I: Yes, regarding this project.**

P: They do not match; there is a difference in quality.

**I: Which one is better, the government’s or KOFIH’s?**

P: KOFIH.

**I: Why do you say KOFIH is better?**

P: I believe I will go back to what I said earlier. They provided training and supported us with tools and equipment. Yes.

**I: Okay. What are your opinions on the efficiency of the project coordinators. How effective do you think they are according to your views?**

P: We can give them a hundred per cent.

**I: Why should it be a hundred per cent, especially considering you mentioned some aspects were not satisfying? Why do you say their efficiency is good?**

P: For instance, even in selecting people for training, someone goes for an extended period. For example, they might take employees for six months, unlike other projects where you might get training for two weeks or one week. You see the difference.

**I: How about the how organizers/ coordinators were responsible in let say, decision-making. How efficient were the project leaders at making quick decisions during the training?**

P: Who?

**I: The project coordinators during the project.**

P: Decisions about?

**I: Any decisions?**

P: They were good at it.

**I: Alright. Regarding the alignment of project activities with the needs, how well did the project complement the needs of healthcare workers during its implementation?**

P: They tried to some extent. Apart from the library issue, which was a significant challenge, we faced other small issues. When we moved to their hostels after leaving the initial hotel, we could find African meals, but upon returning to their hotels, where we were placed in their hostels, even the issue of food became a challenge.

**I: How about the allocation of funds for sustenance?**

P: The sustenance fund allocation was not sufficient. We had to manage within the given budget.

**I: Why do you say it was not sufficient?**

P: It just was not enough.

**I: Alright. What is your feeling on the training content? Did the six months, as you mentioned earlier, provide enough time for comprehensive training?**

P: Not everyone got six months. Some went for six months, others for two months, and some for one month. So, the training varied. When we went, my colleague and I stayed for one month, which was a short time, so what we studied was different from someone who went for six months.

**I: Okay, understood. Let us move on to the impact section. Firstly, do you feel the project has achieved its intended results?**

P: Yes, the project achieved its intended results. For instance, as they aimed to improve maternal and child health by reducing maternal and child mortality rates, it was successful.

**I: Talking about the benefits, besides yourself, to what extent do you think this KOFIH project has strengthened the capacity of other healthcare workers?**

P: Other healthcare workers have benefited from the training we conducted. After receiving training, you are expected to pass on the knowledge. Members who went through the program, as well as others who received training from KOFIH trainers visiting our country, benefited from the internal knowledge transfer.

**I: Can you share other significant changes you have experienced due to your participation in this project, aside from the knowledge you mentioned earlier?**

P: Well, it helped me build connections with other people.

**I: Alright. Let us conclude with the sustainability aspect. Do you believe the achievements of this project, such as the reduction in maternal and child mortality, will continue after the end of the project?**

P: If the funders are not there to support us, I think things will go in a different direction.

**I: Why do you say that?**

P: Our skin cannot thrive without moisturizing.

**I: To ensure sustainability, what aspects do you think should be prioritized if the project’s funder decides to withdraw support?**

P: I think continuing to provide training and eventually handing over the project to the government would be crucial.

**I: Do you believe the government can take over everything initiated by KOFIH and make it sustainable? If so, how?**

P: I believe if the project is handed over to the government and they establish their foundations to ensure continuity, it could be sustained.

**I: Regarding the knowledge you gained from the project, do you think it will be sustainable for you in the years to come?**

P: Yes, because the issue of maternal and child health is in my blood. I acquired the knowledge and continue to implement it, so I don’t think I will reach a point where I stop.

**I: Alright. Thank you very much for your insights.**

P: Thank you.

# **KII WITH KOFIH BENEFICIARY**

| **Date** | 21/11/2023 |
| --- | --- |
| **Region** | Coastal |
| **Location** | Tumbi Referral Hospital |
| **Language used** | Swahili |
| **Translated** | English |
| **Gender** | Female |
| **Position** | RN |

**I: As a beneficiary of this project, I’ve come to talk to you about your experiences with this project. Could you please introduce yourself and briefly explain when you started participating in this project? Then we can proceed with our questions.**

**P:** My name is ………., and I attended the Korean training for three months in 2012 while working in the pediatric department.

**I: Right here at Tumbi?**

**P:** Yes, here at Tumbi, and my current position is Assistant Nurse Officer.

**I: Great, thank you for the introduction. Let’s dive into our questions. First, let’s discuss the relevance of the project. In your opinion, did the project address the needs and priorities of healthcare providers? Let say, the training package or the objectives of the program did it reflect the needs and priorities of healthcare workers?**

**P:** When we started, here at Tumbi, I think my intake was the third one. When Korean people come, they even started to provide us with various equipment for pediatric especially for neonates. Before we did not have equipment such as suction devices and incubators. By that time, they brought many equipment and electronic machines. There was limited utilization of the equipment…so when the fellowship opportunities were announced, we went and it really helped us in utilization of the provided equipment. By then, we benefited a lot since the project had been just introduced to our facility.

**I: Okay...!! Do you think the project’s training package and outcomes align with the needs of the country and your department?**

**P:** Absolutely. The project was beneficial, considering our lack of equipment as I initially mentioned. It provided us with the training and equipment. The project was good because when we come back, we were able to share the gained skills to others. So the project is very good especially for us who participated during that period.

**I: What would you say are the country’s priorities that the project addressed effectively in your department?**

**P:** For me I think the priority is equipment, since this was the major challenge, we faced…even still but at least now they have managed to cover 85 percent of the equipment which we used to lack. In this sense, this is good part of the project that was implemented.

**I: Apart from KOFIH project, were there other projects addressing maternal and child health that you are aware of?**

**P:** Yes, there are others. Although I can’t remember all of them, I’m more familiar with KOFIH. There are also projects from KOICA, but KOFIH is the one we are more accustomed to.

**I: Do you think the KOFIH project complements other health projects in the maternal and child health department?**

**P:** Yes, it does. The projects share the common goal of helping us. Thus, we benefit more from these projects when they complement each other.

**I: Can you give an example of how the KOFIH project complements other maternal and child health projects in your department?**

**P:** Do you mean what program supported…did you meant that?

**I: Yes.**

**P:** It has supported many things not only for RCH services only…but also emergency department where several equipment was provided. Even here at pediatric if you put aside the neonate pediatric, the program also supported. Again, we even have outreach activities for training about biomedical equipment in other regions.

**I: So…you visit other places?**

**P:** We do visit, even the past days we KOFIH facilitated our trip to Shinyanga for providing training.

**I: Okay…so to what extent do you think the program align with the priorities of other stakeholders let’s say with health committees at district and regional level. They [committees] do have their priorities on one way or the other. So how does the project support the priorities of the CHMT and RHMT?**

**P:** Yet. It supports them…we have heard that KOFIH went to meet with CHMT and RHMT. There is other equipment you will find them to those committees for example the laptops your will find they have KOFIH stickers. It is fair to say they discuss and support the health committees.

**I: Okay, do you think the design of this program aimed to address the challenges and needs of healthcare workers?**

**P:** of course…I can say that it has managed to address the challenge, for example like I have said before…by the time I was employed in 1999…there some equipment which did not have…but through KOFIH we have received such equipment.

**I: Besides equipment, what others…!**

**P:** Even the continuous knowledge…these on job training. They provided some workshops at Mlandizi, other regions and health centers. They even go in peripheral areas for training. So, to me, this has helped a lot.

**I: Leave alone the things which were implemented by the program. Maybe what are the major priorities or challenges facing healthcare workers? Those challenges which inhibit the delivery of services among healthcare workers?**

**P:** Infrastructure and logistics are significant challenges. For example, I am working in a referral hospital but when I visit other health centers where they make referral of pediatrics to us…they have a lot of challenges from roads…to equipment, maybe a neonate needs to be brough to referral with oxygen on. There are a lot of challenges. But for us here, KOFIH has supported many equipment…when I receive a referral case, I can manage through the support provided by KOFIH.

**I: Okay…going to effectiveness. Here let’s start with your opinion regarding the training provided. What are your opinions regarding the training provided in Korea? Generally, what are your thoughts?**

**P:** I believe that when there is a continuous training so many people can acquire knowledge and skills. I second for trainings to continue as they are beneficial.

**I: Here I want your experience for example about preparation prior to training sessions…the selection of participants. The process of application, selection of participants…and what happened when you reached there...**

**P:** Should I respond for us when we were going?

**I: Exactly, give response from your own experience.**

**P:** When we were going during that time, they used to come and visit us at wards. Then they make request that they want like two staffs. Then matron or of medical officer in charge select staffs for training. During our intake, four of us were selected.

**I: Using the same procedure?**

**P:** Yes, they chose us. They sent an email saying they needed one nurse and doctor from Tumbi, but a doctor had already gone by that time, and was selected by the same procedure. So, when it came to the nurses, they requested nurses from the pediatric and labor wards. Four were selected, and then we were told we would have interviews individually through a phone call from Korea. After the results came out, I was told I passed, and I was given those forms to fill out what I wanted to learn…I filled the form with medical officer in charge, and I went for three-month program. That was how we were selected, but for now I am not aware on the selection procedures.

**I: Okay..., how do you perceive the training content? How did you view the training package, including the availability of materials and the topics covered?**

**P:** Theoretically, I enjoyed it. Theoretically, I enjoyed it because we were given people who understood English, accompanying us, especially the nurses am not sure about doctors. But practically, it was challenging. You can’t touch the machines; you are just told that this is an incubator, the baby is placed like this and this. So, you had to use your brain a lot to understand where to press or what to do. It was challenging for us to touch their patients.

**I: Is there any specific reason given as to why you couldn’t touch patients?**

**P:** We didn’t ask; we just felt it was their way. We also have a rule that there are some students who shouldn’t touch a baby. So, from my perspective, theoretically, I found it good, if you put personal efforts, you can understand very deep.

**I: Okay. You mentioned the selection process where the matron chose candidates. Do you feel there was gender equality in that process?**

**P:** I don’t know how to put it! There might equality because a male doctor went, and I, a female, went. However, I’m not sure how things were previous

**I: Okay. What are your thoughts on the project’s ability to achieve its goals, especially after the closing phase of the project?**

**P:** what I think I can suggest is that...there was a time it seemed like they stopped by focusing more on training us, then us to go and teach others. From my perspective, looking at my long tenure here I see like the support of equipment and maintenance of equipment has stopped…we no longer see the same trend anymore. They used to come frequently; they would come themselves, inspect their machines, check where there were challenges, and address issues. Now, I don’t see that exercise happening for a long time; I’m not sure.

**I: Why do you say it seems like they stopped in terms of maintenance?**

**P:** I think they stopped because I haven’t seen them in a long time in that regard. During that time, when we started, they would come not more than a month apart. They would come, want to inspect their equipment; you would attend to them with challenging equipment, and they would point out where the problems were. And if the equipment could not be repaired, you would here the medical officer in charge saying that we have received another equipment. I haven’t seen that exercise for a long time now.

**I: Okay. You said you haven’t seen that in a while, so when equipment breaks down, are those trained in biomedical engineering capable of fixing them?**

**P:** Yes, they fix them.

**I: Okay. What is your perspective on the availability of staff? Well, here, you already mentioned that you were selected. However, concerning the reporting aspect, how did that work? You mentioned they come, observe, and then report to the matron that we need these people. How long does it take for the matron to inform you that you need to go or that certain things are required for you to go?**

**P:** I heard that send email to the medical officer in charge. Once he receives the email, he talks to the matron. The matron then calls us, mentioning names if it’s all four of us. She tells us that we will have interviews, fill out forms, and prepare for Korea. So, it was within short period of time.

**I: Okay. Based on estimates, wow long did it took?**

**P:** after receiving email…. for our medical officer in charge, it took about a week.

**I: Within a week, you need to complete everything?**

**P:** Yes, yes! For us, he informs us within a week after receiving the email, and we do interviews a week later. So, it’s swift, as he helped us.

**I: Are those selected two, the only ones filling out the forms and doing those activities?**

**P:** Yes, for those in the past they did it themselves.

**I: Okay. Being among the project participants, can you tell me what it feels like to be part of the project? How do you feel?**

**P:** I feel very good.

**I: Why?**

**P:** I feel very good because I’m still within the project…why I say this….as we speak, I came from Shinyanga through the KOFIH project to train in critical care, meaning I’m still part of the project. So, I feel I’m still within the project.

**I: Okay. Had it been no project such as this, or you didn’t receive this opportunity to attend training, how do you think your situation would be in terms of performance?**

**P:** Even if we hadn’t received those equipment…or going for training, then we wouldn’t be this far as we are now. but after receiving the equipment and went for training, it means we have benefited that is why we are enjoying the current state.

**I: Okay, let’s move to the third section which is about efficiency. Here, I have a few questions. Firstly, can you tell me if the project activities interfere with the implementation of other projects?**

**P:** Yes

**I: Are there any areas of convergence between KOFIH project activities and other projects? Any overlapping or conflicting activities?**

**P:** Do you mean like another project which complement implementation of one thing or inhibit another thing?

**I: No, I mean, KOFIH offers training and equipment, isn’t it? So, are there other projects doing the same thing, like providing the equipment?**

**P:** As far as I know, KOFIH and the Ministry of Health are the ones currently bringing equipment and training.

**I: Do they also align with KOFIH’s training package, or are they different?**

**P:** They align because what we went to teach is from the Ministry of Health. It’s from the Ministry, but KOFIH facilitated it. So, they work together.

**I: They work together, and these are things that are similar?**

**P:** Yes.

**I: Okay. Could you talk about the efficiency of the training provided through this KOFIH fellowship programme? How about the content and topics covered in terms of empowering you with new knowledge and skills?**

**P:** They provided us with excellent efficiency because we often attended conferences, meetings, and went to practical areas. It was well organized; we received books and materials.

**I: So, the materials were readily available?**

**P:** Yes, they were available.

**I: Was there any difficulty, maybe language issues on the available material?**

**P:** Regarding language, no any difficulty in material availability. They were very organized. As for language maybe outside the classes, but we were given someone who escorted us and was knew English effectively. So, we could communicate with them most of the time. The only challenge was when we were outside the hospital. But otherwise, we had no issues.

**I: About the stipend, were you paid on time? Were the activities being done timely?**

**P:** We were paid on time, and we had an account where the money was deposited on time.

**I: Okay, were the topics covered on time?**

**P:** It was timely because even if we worked the whole day, in the evening, we would write reports and send them to our immediate supervisors.

**I: Let’s move to the fourth section, which is about impact. Do you feel like the project has achieved the intended impact?**

**P:** Without a doubt, from my perspective, it was very successful.

**I: Why?**

**P:** Because, as I mentioned, we were the first beneficiaries who went when the hospital wasn’t in its best condition. When they brought the equipment, our colleagues who had returned started using one after the other, and we began our work. During that time, we hadn’t initiated the NICU yet, so when we returned, we started thinking about establishing it. We were given additional NICU equipment. We had our equipment, so the impact was good, especially during that time.

**I: All you said is about that time, how about now?**

**P:** It’s still the same; we continue. However, there are times when it seems like they stopped or are continuing in some other areas. But during that time, we saw them often, especially regarding our equipment. However, at some point, it seemed like it stopped.

**I: Okay. What do you consider as barriers that could hinder the achievement of the mentioned positive outcomes?**

**P:** Barriers?

**I: Mmmh! Maybe any challenges that might inhibit those good things you mentioned?**

**P:** There aren’t. We continue using what we have gained, and if there is any challenge with equipment, there is someone who was trained, they came and fixed them.

**I: Okay. Can you tell me how this project benefited you?**

**P:** I’ve benefited a lot educationally—knowledge, and enough skills to change my environment. I’ve also seen what our colleagues are doing and what we should be doing. I’ve personally changed a lot of things.

**I: What about the execution of your duties?**

**P:** As Linda, I execute them well because I possess a sufficient package. I have not forgotten, even though it has been a while since I went, I have not forgotten the things which I learned during my time there. I still know and apply them, even sharing them with my colleagues. For example, they may request you to support them for certain things simply because you went to Korea. So, you have to show them the knowledge and skills that you gained.

**I: Okay, good. Regarding your experience, are there any changes you see in you because of participation in this program? Put aside what you have shared related to knowledge, and skills, are there any other major changes that you see?**

**P:** To me?

**I: Any, which you think you have done well.**

**P:** I’ve changed in terms of ethics. I’ve gained more knowledge and skills. Even if I go outside, my colleagues can differentiate me and appreciate that “I went to Korea”. If they ask me to do something, I will do it. So, I feel different; I feel unique because I went, got trained, and came back a different person.

**I: Okay…okay. What do you consider to be unintended impacts of the project? I know you have shared with me many impacts, maybe if we put aside those impacts related to new knowledge and skills you acquired and equipment provided during the training, what other impacts do you think are resulting from the project activities? Can be positive or negative.**

**P:** I don’t know what to say, but I see…

**I: So, we’re talking about the unintended impacts of the projects. I want to know if there are any impacts that came out of the project but they were not within the intention of the project. For instance, if the project planned to provide knowledge, but upon arriving, you found other things apart from knowledge. Or if the project aimed to supply equipment, but besides the equipment, the hospital obtained other things not initially targeted by the project.**

**P:** I can say there are unintended impacts.

**I: Can you mention some?**

**P:** Yes, there are many because I remember when we went, it wasn’t during the alumni time. We had just returned from Korea, but there was a period when we could go through KOFIH and request to go provide services to the poor in rural areas who didn’t have access to equipment. For example, we wanted to go and conduct specific tests for pregnant women. I think we would write a request, and then a Korean lady would come, and we would go with her to those places and provide services. It wasn’t part of the project, but they helped us by connecting us. They would go there themselves, pick us up, and take us. I think this wasn’t part of the project, but we still went.

**I: What else?**

things I gained personally, but for the project, I think it has already fulfilled itself.

**I: Okay. Let’s conclude with the sustainability aspect of the project. Suppose, KOFIH says, now we stop funding the project. Do you think the positive results of the project will still be sustainable?**

**P:** Of course, we’ll try. For example, if I went in 2012 and I’m still doing things in 2023, and even when the Koreans come asking about how we use the equipment, how we manage certain things, we can answer. I still can go and teach others what I took from Korea. It means we can try to continue unless there’s a significant change.

**I: Okay.**

**P:** Yes.

**I: Based on your experience, what do you think should be given priority by the project to ensure sustainability even if the project comes to an end?**

**P:** They should provide us with more equipment, even if we already have some. Also, they should train more biomedical staff to assist us. It would be good if they could increase the number of biomedical staff to support us at all times regarding equipment issues.

**I: Currently, aren’t they available all the time?**

**P:** There are, but not those who went to Korea.

**I: Okay.**

**P:** There are normal biomedicals.

**I: Anything else that should be given priority?**

**P:** Another thing is facilitating us to continue going to teach others. We should continue going to our colleagues to provide training and support because we see the need. If we find a place in need, we request to go and help. We need to maintain that because it’s beneficial.

**I: Okay. What about you, personally? Do you think you will continue… I know you’ve been using this for over ten years now. Do you think other participants of KOFIH will continue to use the knowledge and skills gained in the course of executing their duties?**

**P:** Individuals differ a lot, so I can say they might or might not. But speaking for myself, I am proud of it because I’ve done justice to it, and that’s why I’m still here. If others come back and find us, we will remind each other to continue maintaining the positive impact.

**I: Is there anything you can advise other participants who have received this training on how to ensure the skills and knowledge they gained remain sustainable?**

**P:** Of course, they should join the alumni because, by joining, we all benefit, not just those in the group. We all go to teach, write abstracts, and get opportunities. By staying there, we remind each other. We have close communication even through WhatsApp. We meet a lot to prepare for events, and there is constant communication.

**I: How many times do you meet in a year?**

**P:** Twice a year, or more if there are travels. We meet often for preparations, and WhatsApp communication is frequent.

**I: You said they should join alumni groups, aren’t they all on these groups?**

**P:** I don’t know; some might not have WhatsApp. But everyone should join the alumni. I think some might not be there, but in my opinion, they should join to maintain what we have.

**I: What else should be a priority?**

**P:** They should provide more of the equipment we already have and train more biomedical staff. Also, they should facilitate us to continue teaching. That would be good for maintaining what we have.

**I: Okay, what about other institutions like the government ones? Do you think they can take over the KOFIH project and continue to implement the project activities and outcomes when KOFIH decides to pull off providing the support?**

**P:** It is very possible through us…beneficiaries who went there.

**I: No, apart from you…beneficiaries, how about the government and other health stakeholders?**

**P:** It is possible

**I: How is it possible?**

**P:** Yes, if there is a need for it. Based on my understanding, there is the Ministry of Health …so if the program pulls out, the ministry is there and will take over the implementation. These programs like KOFIH are just complimenting, if they continue it will be good, but if they pull off, then the Ministry of Health and other stakeholders will continue.

**I: Do you think, the Ministry of Health can assume the role of KOFIH?**

**P:** Yes, it can, because…

**I: What is needed?**

**P:** Eeh

**I: You are not sure?**

**P:** I am…but I think it must be able for a hundred percent. From what I see even these stakeholders are just coming to compliment…we appreciate their contribution. But we cannot say that if they pull off their support then we cannot deliver at all. Does it mean that if not for them, then we could not have done anything? But we could have managed.

**I: Okay, do you have any additional comments you’d like me to hear about the KOFIH project before we conclude the interview?**

**P:** I don’t have any; I just want to thank you because what we’re doing might help those in management to take feedback and see where to improve, what to reduce, and what to increase.

**I: Well, I appreciate. Until this point, we have reached the end of our interview. Thank you very much.**
