# Supplementary material for: Empirical effect of the Dr LEE Jong-wook Fellowship Program to empower sustainable change for the health workforce in Tanzania: a mixed-methods study
Source: J Educ Eval Health Prof. 2025 Jan 20;22:6. doi: 10.3352/jeehp.2025.22.6 (PMC12003955; doi:10.3352/jeehp.2025.22.6)
Supplement: Supplementary file 3 — Supplement 1. Quantitative and qualitative questionnaires. [file jeehp-22-6-suppl1.docx]

**Online Survey Questionnaire**

**Introduction**

As part of the continuous improvement strategy of its healthcare programs, and with the intention of enhancing healthcare sector in Tanzania, KOFIH Tanzania Office periodically evaluates its projects and programmes. On this occasion, KOFIH Tanzania Office is evaluating the ‘Dr LJW Fellowship Program’ which started in 2009 and has been conducted yearly with the aim to nurture experts who are expected to play a leading role in healthcare improvement.

The KOFIH records show that in the period ranging from 2009 to 2022, you were among the healthcare workers who have participated in the program. We therefore request you to respond to the attached survey questionnaire, in order to gauge your perceptions on the quality and usefulness of the Dr LJW Fellowship Program. Your cooperation would be highly appreciated. The survey will be confidential, and we anticipate it will take about 15 minutes of your time. The evaluation team would appreciate if the responses to questionnaire would be sent back to us as soon as possible. Your opinions will be handled with strict confidentiality and will be very useful to improve the programme.

**A1. REGION**

___________________________________________

**A2. INSTITUTION (EMPLOYER)**

__________________________________________

**A3. DATE**

**A4. Respondent’s Name (OPTIONAL)**

_________________________________

**A5. Respondent’s Age**

__________________________________________

**A6. Respondent’s Department**

__________________________________________

**A7. Sex of Respondent**

1. Male
2. Female

**A8.** Please mention the institution/organization you worked for at the time you were participating in the Dr LJW Invitational Fellowship Program.

__________________________________________________

**A9.** Are you still working with the same institution/organization?

1. Yes
2. No

**A10.** Which of the following training activities (courses) did you attend? (i.e. through the Dr LJW Invitational Fellowship Program)

1. Clinical Practices
2. Biomedical Engineering
3. Health Administration
4. Infectious Disease Control
5. High Level Official Training
6. Other (please specify) __________________

**A11.** Please mention the year of your participation in the course above: ________________________

**RELEVANCE**

**B1.** Why did you participate in the training activity (course) you identified/mentioned above through Dr LJW Invitational Fellowship Program?

1- to explore my personal interests

2 – because my supervisor/head asked me to attend

3 – to gain extra skills for my current job/work

4 – to get a different job within the institution/organization I am currently working for

5 – to get a new job in another institution, organization etc.

6 – to grab the chance to go abroad

7 – to network with other participants in the programme

8- Other (please specify) __________________

**B2.** Is the content of the training activity (course) still relevant for your current function/work?

1- Not relevant at all

2 – Not relevant

3 – Neutral

4 – Relevant

5 – Very relevant

**B3.** The overall training through Dr LJW Invitational Fellowship Program was beneficial to your work:

1- Strongly disagree

2 – Disagree

3 – Neutral

4 – Agree

5 – Strongly agree

Please explain your answer in B3: **________________________________**

**B4.** Were the goals of Dr LJW Invitational Fellowship Program provided/communicated to you before attending the programme?

1- Yes

2- No

**B5.** The trainers/facilitators were helpful in guiding you to understand the training material you were provided while attending the programme:

1 - Strongly disagree

2 – Disagree

3 – Neither agree nor disagree

4 – Agree

5 – Strongly agree

**B6.** Is there any specific knowledge you have used on your work after participating in the Dr LJW Fellowship Program?

1- Yes

2- No

If yes, please explain what knowledge and how you have used it.

______________________________________________________________________________

**B7.** Is there anything which has changed your perception, attitude or behaviour as a result of participating in the Dr LJW Fellowship Program?

1- Yes

2- No

If yes, please give an example.

______________________________________________________________________________

**B8.** How relevant were the contents of the training in which you participated to the country needs and priorities?

1- Not relevant at all

2 – Not relevant

3 – Neutral

4 – Relevant

5 – Very relevant

**B9.** Was the training relevant to your work needs and priorities?

1- Not relevant at all

2 – Not relevant

3 – Neutral

4 – Relevant

5 – Very relevant

**EFFECTIVENESS**

**C1A.** To what degree did the Dr LJW Fellowship Program satisfy/live up to your initial expectations in terms of quality of the substantive content/ topics presented and discussed during the training?

1- Very low

2 – Low

3 – Moderate

4 – High

5 – Very high

**C1B.** To what degree did the Dr LJW Fellowship Program satisfy/live up to your initial expectations in terms of quality of the materials used during the training?

1- Very low

2 – Low

3 – Moderate

4 – High

5 – Very high

**C2.** How useful is the training for the work of your current institution (employer)?

1- Very low

2 – Low

3 – Moderate

4 – High

5 – Very high

**C3.** Your participation in the Dr LJW Fellowship Program contributed to increasing your knowledge and understanding of healthcare issues:

1- Strongly disagree

2 – Disagree

3 – Neutral

4 – Agree

5 – Strongly agree

**C4.** To what extent is the knowledge acquired through your participation in the Dr LJW Fellowship Program useful in your daily work?

1- Not useful at all

2 – Slightly useful

3 – Moderately useful

4 – Very useful

5 – Extremely useful

**C5.** How effective was the program in creating synergies/cooperation among beneficiaries of the Dr LJW Fellowship Program?

1 - Not effective at all

2 – Slightly effective

3 - Moderately effective

4 - Very effective

5 – Extremely effective

**C6.** How would you rate the effectiveness of the training you received through Dr LJW Fellowship Program in helping you and other participants to develop a common understanding of the healthcare issues in the country?

1 – Not effective at all

2 - Slightly effective

3 – Moderately effective

4 - Very effective

5 - Extremely effective

**EFFICIENCY**

**To what extent do you agree with the following statements:**

**D1A.** Maintenance allowance was provided on time by Dr Lee Jong-wook Invitational Fellowship Program.

1 - Strongly disagree

2 - Disagree

3 - Neutral

4 - Agree

5 - Strongly agree

**D1B.** Training materials were provided on time by Dr Lee Jong-wook Invitational Fellowship Program.

1 -Strongly disagree

2 - Disagree

3 - Neutral

4 - Agree

5 - Strongly agree

**D2A.** The application process for applicants to the Dr LJW Invitational Fellowship Program was transparent and consistent.

1- Strongly disagree

2 – Disagree

3 – Neutral

4 – Agree

5 – Strongly agree

**D2B.** The selection criteria for applicants to the Dr LJW Invitational Fellowship Program was transparent and consistent.

1- Strongly disagree

2 – Disagree

3 – Neutral

4 – Agree

5 – Strongly agree

**D3A. T**he application process for applicants to the Dr LJW Invitational Fellowship Program must be reviewed.

1 - Strongly disagree

2 - Disagree

3 - Neutral

4 - Agree

5 - Strongly agree

**D3B. T**he selection criteria for applicants to the Dr LJW Invitational Fellowship Program must be reviewed.

1 - Strongly disagree

2 - Disagree

3 - Neutral

4 - Agree

5 - Strongly agree

**D4.** You were given all necessary information or help during your application, selection, and participation to the program from the coordinators/officers overseeing the Dr LJW Invitational Fellowship Program in the country.

1- Strongly disagree

2 – Disagree

3 – Neutral

4 – Agree

5 – Strongly agree

**D5.** Were the important due dates/time frames for the training program communicated to you before attending the program?

1 - Yes

2 - No

**IMPACT**

**E1. To what extent do you agree with the following statements:**

1. Dr Lee Jong-wook Invitational Training Fellowship Program has increased my motivation to work:

1 - Strongly disagree

2 - Disagree

3 - Neutral

4 - Agree

5 - Strongly agree

1. Dr Lee Jong-wook Invitational Training Fellowship Program has improved my confidence at work:

1 - Strongly disagree

2 - Disagree

3 - Neutral

4 - Agree

5 - Strongly agree

1. Dr Lee Jong-wook Invitational Training Fellowship Program has helped me perform better at work:

1 - Strongly disagree

2 - Disagree

3 - Neutral

4 - Agree

5 - Strongly agree

1. Dr Lee Jong-wook Invitational Training Fellowship Program has helped me translate theory into practice:

1 - Strongly disagree

2 - Disagree

3 - Neutral

4 - Agree

5 - Strongly agree

1. My participation in the Fellowship Program has allowed me to apply the knowledge I got to my work setting and other related healthcare activities:

1 - Strongly disagree

2 - Disagree

3 - Neutral

4 - Agree

5 - Strongly agree

E**2.** To what extent did your competence and on-job performance improve as a result of your participation in the Fellowship Program?

1 – Not improved at all

2 – Slightly improved

3 – Moderately improved

4 – Significantly improved

5 – Highly improved

**E3.** How would you rate Dr LJW Invitational Fellowship Program’s contribution in enhancing the capacity of healthcare workers in the country?

1 - Very low

2 - Low

3 - Moderate

4 - High

5 - Very high

**E4.** To what extent did this training improve your knowledge or strategies to strengthen your institution/organization?

1 – No improvement at all

2 – Slightly improved

3 - Moderately improved

4 – Significantly improved

5 – Extremely improved

**E5.** To what extent did the content of training activity meet your professional needs?

1 - Not likely at all

2 – Slightly likely

3 - Moderately likely

4 – Very likely

5 – Extremely likely

**E6.** By being one of the participants in the Dr LJW Fellowship Program has helped you to develop a sense of collaboration with other participants.

1 - Strongly disagree

2 - Disagree

3 - Neutral

4 - Agree

5 - Strongly agree

**SUSTAINABILITY**

**F1.** To what extent do you think that the program outputs/results would be sustained after the Dr LJW Fellowship Program activities comes to an end?

1- Not likely at all

2 – Slightly likely

3 – Moderately likely

4 – Very likely

5 – Extremely likely

**F2.** To what extent do you think that the programme outputs/results would be sustained by the beneficiaries if the Dr LJW Fellowship Program activities comes to an end?

1- Not likely at all

2 – Slightly likely

3 – Moderately likely

4 – Very likely

5 – Extremely likely

**F3.** Would you recommend your colleagues who are working in the healthcare sector to participate in the Dr Lee Jong-wook Fellowship Program?

1 - Yes

2 – No

**F4.** Please indicate your level of agreement with the statement that there is a potential for the Dr Lee Jong-wook Fellowship Program to be integrated in the national health system strengthening strategies.

1 - Strongly disagree

2 - Disagree

3 - Neutral

4 - Agree

5 - Strongly agree

## In-depth Interview (IDI) Guide

Introduction

My name is ________ from the University of Dar es Salaam. We are currently conducting the Impact Evaluation of Dr Lee Jong-wook Fellowship Invitational Training Program for Healthcare Workers in Tanzania. The project is being implemented by Korea Foundation for International Healthcare (KOFIH). You might have heard about this program before. The program trains healthcare professionals through invitational training programs for healthcare workers. The invitational training program targets clinical experts, health administrators, high-level officials, biomedical engineers, and infectious disease specialists. In this context, we would like to hear your opinion. The purpose of this evaluation is to examine how the program was implemented and what should be done differently in the future. I am hereby requesting your participation in this interview. I would also like to know if you accept that I record the conversation to be able to document exactly what you are saying. Your personal details will remain confidential and your name will not be disclosed in the report. This discussion should take between 45 to 60 minutes.

**A: IDENTIFICATION**

A1. Name of the institution……………………………………………………………………………………….

A2. Contact Person and Position………………………………………………………………………………………….

A3. Region………………………………………………………………………………………

A4. Date of interview…………………………………………………………………………...

A5. Name of interviewer………………………………………………………………………..

**Part 1: Relevance**

1. To what extent are Dr LJW Fellowship Program activities and objectives designed to respond to the needs and priorities of healthcare workers? (Probe if they target the relevant actors). Has the invitational training programme targeted the right beneficiaries? If no, who should be targeted?
2. To what extent has the Dr LJW Fellowship Program ensured that the various needs of healthcare workers are taken into account in both the planning and implementation phases?
3. How do the programme activities and outputs align with and support the country’s priorities?
4. How well does the programme complement and fit with other health related programmes in the country? (probe: links at the district/regional level and facility level)
5. How well does the programme complement and link to activities of other stakeholders in the country? (probe: links at the district/regional level and facility level)
6. To what extent has the programme been relevant to stakeholders’ needs and priorities? (probe: needs and priorities of individual healthcare workers, Regional Health Management Team (RHMTs), Council Health Management Team (CHMTs), and Hospital Management Team (HMTs etc.)
7. Do you think that the Dr LJW Fellowship program design is addressing the most important challenges and needs of healthcare workers? Why or why not?

**Part 2: Effectiveness**

1. How would you comment on the invitational training programme? (probe: organization, recruitment/selection, logistics, training content and delivery, any missing activities?)
2. How has programme promoted equal access to activities, resources, services and skills for both male and female healthcare workers?
3. How would you comment on the extent to which the programme is making sufficient progress towards its planned objectives? Is the programme likely to achieve its planned objectives upon completion?
4. How do you perceive the process of recruiting healthcare workers for the Dr LJW Fellowship Program? (probe: inclusiveness, transparency i.e. who is involved, flow of information etc.)
5. How did the acquired training through the Dr LJW Fellowship Program meet the expectations of the targeted beneficiaries (participants)? (Probe: aspects that were satisfied, disappointments, wishes etc.)
6. Could you tell me what it was like to take part in the Dr LJW Fellowship Program? (For those who went for training)
7. How do you think things would have been, if you would have not participated in the programme? (For those who went for training)

**Part 3: Efficiency**

- - 1. Do Dr LJW Fellowship Program’s activities overlap and duplicate other similar interventions that are funded nationally and /or by other stakeholders?
    2. How efficient are the management and accountability structures of the programme? (probe: time taken to make decisions)
    3. To what extent does the allocation of resources reflect the needs of healthcare workers?
    4. Have programme funds and activities been delivered in a timely manner? (probe: delays & complaints)

**Part 4: Impact**

1. Has the programme achieved the expected results? (Probe if there are any barriers that prevent these results from being achieved).
2. How have you benefited from this programme? (probe: type of support, duration of support, changes as a result of the support i.e. performance, realizing your potential to serve the community, value added to the community etc.)
3. To what extent has the programme strengthened the skills of healthcare workers? (Probe how from previous experiences).
4. How are capacities strengthened at the individual and organizational level?
5. What is the contribution of the programme to quality of health service delivery in the programme areas?
6. What is the most significant change you have experienced as a result of your participation in the programme?
7. Is there unintended impact of the programme? (Probe for positive/negative impact).

**Part 5: Sustainability**

1. Do you think the current achievements from the programme will have a long-term impact even if there is no more training and support from KOFIH? (Probe to what extent the benefits of Dr LJW Fellowship Program are likely to be sustained after the completion of the programme).
2. What are the key factors that will require attention in order to improve prospects of sustainability of Dr LJW Fellowship Program outcomes? (Probe for the main lessons that have been learnt).
3. Do you think you are going to use the skills you acquired in the long-run?
4. Once external funding ends will government institutions and implementing partners be likely to continue the project or carry forward its results?
5. How regular do you attend the meetings with other alumni?
