# Supplementary material for: Empirical effect of the Dr LEE Jong-wook Fellowship Program to empower sustainable change for the health workforce in Tanzania: a mixed-methods study
Source: J Educ Eval Health Prof. 2025 Jan 20;22:6. doi: 10.3352/jeehp.2025.22.6 (PMC12003955; doi:10.3352/jeehp.2025.22.6)
Supplement: Supplementary file 4 — Supplement 2. The cumulative number of participants in the Fellowship Program. [file jeehp-22-6-suppl2.docx]

Supplement 2. Total Cumulative Number of the Fellowship Program’ Participants from 2009 to 2022

| **Year** | **Course’s Name** | **Region** | **Cumulative N (=165) (M:F)** |
| --- | --- | --- | --- |
| 2009 | Clinical Expert | Pwani | 3 (3:0) |
| 2010 | Clinical Expert | Dar es Salaam | 5 (1:4) |
|  |  | Pwani | 2 (1:1) |
|  | Health Policy & Administration | Dodoma | 2 (2:0) |
|  | Biomedical Engineer | Pwani | 2 (2:0) |
| 2011 | Clinical Expert | Dar es Salaam | 2 (2:0) |
|  |  | Pwani | 3 (2:1) |
|  | Biomedical Engineer | Dar es Salaam | 2 (1:1) |
| 2012 | Clinical Expert | Dar es Salaam | 8 (2:6) |
|  | Biomedical Engineer | Dar es Salaam | 2 (2:0) |
| 2013 | Clinical Expert | Dar es Salaam | 8 (6:2) |
|  | Biomedical Engineer | Dar es Salaam | 4 (2:2) |
|  |  | Dodoma | 2 (2:0) |
| 2014 | Clinical Expert | Dar es Salaam | 7 (3:4) |
|  | Health Policy & Administration | Dar es Salaam | 2 (1:1) |
|  |  | Njombe | 1 (1:0) |
|  | Biomedical Engineer | Dar es Salaam | 4 (3:1) |
| 2015 | Clinical Expert | Dar es Salaam | 7 (7:0) |
|  | Health Policy & Administration | Dodoma | 2 (2:0) |
|  | Biomedical Engineer | Mwanza | 2 (2:0) |
| 2016 | Clinical Expert | Dar es Salaam | 9 (5:4) |
|  | Health Policy & Administration | Dodoma | 2 (1:1) |
|  | Biomedical Engineer | Mwanza | 5 (5:0) |
| 2017 | Clinical Expert | Dar es Salaam | 7 (4:3) |
|  |  | Pwani | 1 (0:1) |
|  | Health Policy & Administration | Dar es Salaam | 1 (0:1) |
|  |  | Dodoma | 1 (0:1) |
|  | Biomedical Engineer | Mwanza | 4 (2:2) |
|  | High-Level Official | Dodoma | 2 (2:0) |
| 2018 | Clinical Expert | Dar es Salaam | 4 (3:1) |
|  | Health Policy & Administration | Kilimanjaro | 1 (1:0) |
|  | Biomedical Engineer | Dar es Salaam | 4 (2:2) |
| 2019 | Clinical Expert | Dar es Salaam | 7 (5:2) |
|  | Health Policy & Administration | Mbeya | 2 (1:1) |
|  | Biomedical Engineer | Dar es Salaam | 2 (1:1) |
|  | High-Level Official | Dodoma | 1 (1:0) |
| 2020 | Clinical Expert | Dar es Salaam | 4 (4:0) |
|  | Health Policy & Administration | Arusha | 1 (1:0) |
|  | Biomedical Engineer | Singida | 1 (1:0) |
| 2021 | Clinical Expert | Dar es Salaam | 4 (1:3) |
|  |  | Pwani | 2 (0:2) |
|  | Health Policy & Administration | Morogoro | 1 (0:1) |
|  | Biomedical Engineer | Dar es Salaam | 3 (2:1) |
|  |  | Dodoma | 1 (1:0) |
|  | Infectious Diseases | Dar es Salaam | 7 (4:3) |
| 2022 | Clinical Expert | Dar es Salaam | 1 (0:1) |
|  |  | Dodoma | 2 (2:0) |
|  |  | Mbeya | 1 (0:1) |
|  | Health Policy & Administration | Dodoma | 2 (0:2) |
|  | Biomedical Engineer | Dar es Salaam | 2 (1:1) |
|  | High-Level Official | Dodoma | 1 (1:0) |
|  | Infectious Diseases | Dar es Salaam | 3 (3:0) |
|  |  | Dodoma | 2 (1:1) |
|  |  | Kilimanjaro | 2 (2:0) |
|  |  | Songwe | 1 (1:0) |
|  | Master’s Degree | Dar es Salaam | 1 (1:0) |
